# Adsorption Studies at the Graphene Oxide–Liquid Interface: A Molecular Dynamics Study

**DOI:** 10.1021/acs.jpcc.2c07080

**Published:** 2023-03-20

**Authors:** Visal Subasinghege Don, Lukas Kim, Rolf David, Julia A. Nauman, Revati Kumar

**Affiliations:** † Department of Chemistry, Louisiana State University, Baton Rouge, Louisiana 70803-1804, United States

## Abstract

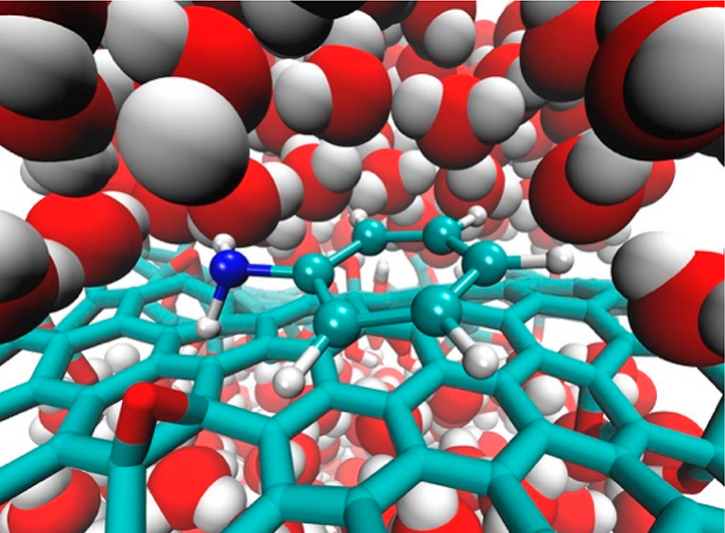

The adsorption of organic aromatic molecules, namely aniline, onto graphene oxide is investigated using molecular simulations. The effect of the oxidation level of the graphene oxide sheet as well as the presence of two different halide salts, sodium chloride and sodium iodide, were examined. The aniline molecule in the more-reduced graphene oxide case, in the absence of added salt, showed a slightly greater affinity for the graphene oxide–water interface as compared to the oxidized form. The presence of the iodide ion increased the affinity of the aniline molecule in the reduced case but had the opposite effect for the more-oxidized form. The effect of oxidation and added salt on the interfacial water layer was also examined.

## 1 Introduction

The contamination of groundwater reservoirs and human wastewater with environmental pollutants is one of the major crises around the world. Carried through industrial effluents and agricultural runoff, chemical pollutants are released into groundwater reservoirs and freshwater sources, exposing animals as well as humans to potentially toxic chemicals. The release of organic dyes, heavy metals, pesticides, and herbicides into freshwater sources poses a threat to communities and ecosystems around the world, particularly in regions where freshwater sources are scarce.^1^ Many physical, biological, and chemical water purification methods are employed in the remediation of groundwater and the treatment of human wastewater. Adsorption is one of the most common techniques for removing pollutants and is used for water remediation in wastewater treatment.^2^ Adsorbent materials, such as activated carbon, biochar, and carbon nanotubes, passively filter wastewater effluents by adsorbing heavy metals and organic pollutants to their surfaces. Recently, graphene oxide (GO) has emerged as a promising adsorbent material with enhanced adsorption of environmental pollutants due to its high tunability and cost-effectiveness for mass production.^2^

GO can range from a single layer to multiple layers and is composed of graphene sheets modified with oxygen functional groups such as epoxy, hydroxyl, ether, carbonyl, sulfate, and sulfonate groups.^3,4^ The GO material contains sp^3^ carbon atoms which are bound to the oxygen functional groups and sp^2^ carbon atoms which represent the graphene part of the material. Due to the existence of these oxygen functional groups, the GO material has both hydrophobic as well as hydrophilic domains.^5^ Interactions such as hydrogen bonding, π–π stacking, and van der Waals interactions govern the structure and dynamics of the GO–aqueous interface. Surface interactions as well as the hydrophilic and hydrophobic nature of the GO sheet can be fine-tuned through modification of the number and type of oxygen-bearing groups on the surface. The high tunability of the GO sheet makes it a suitable candidate for several applications. Water purification, gas separation membranes, electrodes in energy storage devices, and proton conducting membranes in fuel cells are a few of such applications of the GO material.^6−10^ In recent years, GO materials have been applied as an adsorbent material to adsorb organic contaminants as well as metal ions from aqueous phases.^11−13^ Work by Carr et al., using a combination of X-ray reflectivity and sum frequency generation spectroscopy to study the adsorption of yttrium ions on graphene oxide systems, has shed light on the importance of ion solvation and the ordering of water on the ion adsorption process, emphasizing the importance of nanoscale investigations.^14^ Although experimental studies with GO have been conducted on the adsorption of toxic material from industrial waste,^13,15^ current molecular-level insights on the interactions that govern these chemical processes are lacking. A molecular-level understanding of the GO–aqueous interface can enable further optimization of the adsorption process.

The goal of this study is to use molecular modeling methods to obtain molecular-level information on the toxic material adsorption process using GO as the adsorbent. Here, an organic contaminant molecule, namely, aniline, has been studied as a prototypical toxic aromatic compound. Aniline is a highly toxic organic compound that is found in wastewater.^16^ This compound is extensively used in various industries such as the manufacturing of pigments, dyes, herbicides, etc., and can ultimately end up in pure water reservoirs. In this study, molecular dynamics simulations and enhanced sampling methods were used to gain an in-depth understanding of the adsorption of aniline-like organic contaminant molecules at a molecular level. The study focuses on finding computational models that can accurately capture the correct structure and dynamics of the GO–aqueous interface. The effects of ionic strength, the oxidation level of the GO material, and crucial molecular interactions that are the driving forces for the adsorption of aniline onto the GO surface are examined. The insights obtained through these studies will aid in the optimization of the adsorption process and the development of effective wastewater treatment methods using GO. The paper is divided into four sections. In Section 2, the methods used in the paper are outlined. The results and discussion are presented in Section 3, while the conclusions are presented in Section 4.

## 2 Methods

### 2.1 System Definitions and Equilibration

In total, six systems were created to study the effects of salt ions (pure water, NaI, NaCl) and oxidation levels (GO_2/1_, GO_4/1_) on the adsorption of aniline to the GO sheet. The details of the system setup and equilibration are described in this section.

**Figure 1:**
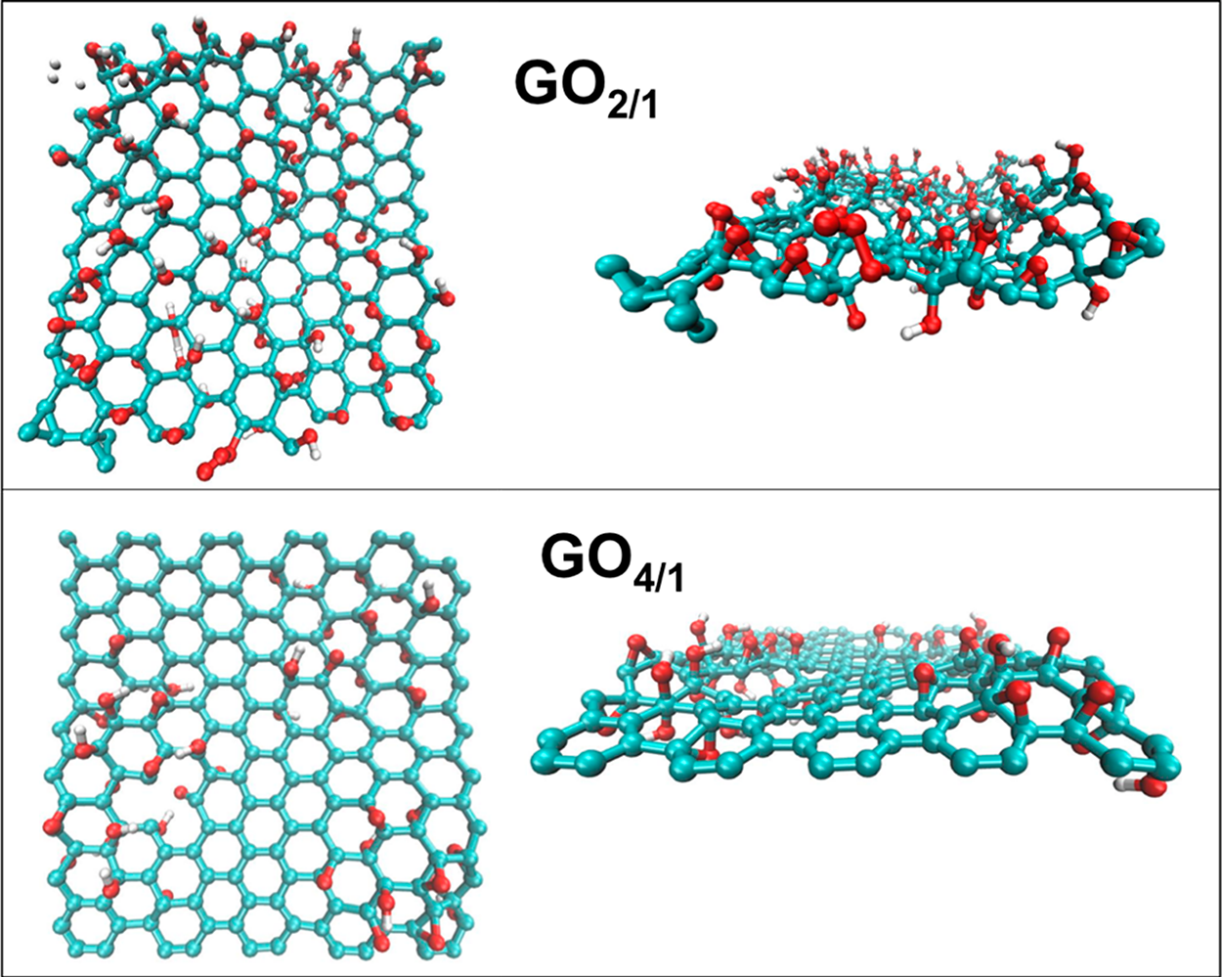
Top and side views of the GO_2/1_ and GO_4/1_ sheets used in the simulations.

The two GO sheets, referred to as GO_2/1_ and GO_4/1_, were built with carbon-to-oxygen ratios of 2:1 and 4:1, respectively. Both GO sheets were composed of a single layer of 180 carbon atoms with oxygen-bearing functional groups on each side. The oxygen-bearing functional groups on the GO_2/1_ sheet consist of 50 epoxy and 40 hydroxyl groups, totaling 90 oxygen atoms. The functionalization of the GO_4/1_ sheet consists of 24 epoxy and 20 hydroxyl groups, totaling 44 oxygen atoms. The GO sheets were created based on the work by Sinclair et al.^17^ and has been used in previous studies by David et al.^18,19^ The sheets were then subjected to an energy minimization followed by short 1 ns simulations in the NPT ensemble (*T* = 300 K and *P* = 1 bar). The force-field and simulation parameters are discussed in the following section. The optimized sheets are shown in Figure 1. Using the software Packmol,^20^ the resulting GO_4/1_ sheet was solvated with 1570 water molecules, and the GO_2/1_ sheet was solvated with 1757 water molecules. The initial dimensions of the systems were approximately 21 × 21 × 125 Å^3^. This effectively results in a system with GO sheets approximately 100 Å apart, thereby minimizing the interaction between sheets. This is a reasonable approximation for a monolayer graphene sheet–water system.

The water molecules were represented using the SPC/E^21^ water model. The three-body Tersoff potential^22^ was used to model the graphene part of the GO sheet. The Tersoff potential parameters for the graphene part were taken from the literature.^23^ The OPLS-AA^24^ force-field was used to model the bonded terms of the oxygen functional groups of the GO sheet as well as to model the nonbonded interactions of the water molecules with the sheet. The nonbonded interactions and the charges of the components of the GO sheets were taken from the literature.^25^ The charge of the salt ions was scaled by 0.8 as has been done previously in the literature to take into account electronic polarization effects in a mean field manner.^26,27^ Simulations were carried out under periodic boundary conditions with the long-range electrostatic interactions modeled using the PPPM (particle–particle particle–mesh) Ewald method.^28^ The Lennard-Jones interactions were cut off beyond 10 Å. In order to constrain the bonds and angles of the water molecules, the SHAKE^29^ algorithm was used. All the simulations were carried out using the LAMMPS^30^ package.

The initial energy minimization was followed by a simulation in the NVT ensemble for 10 ns with a 1 fs time step. The temperature of the system was maintained at 300 K using a Nosé–Hoover thermostat.^31,32^ Following this, the system was equilibrated for a further 5 ns with a time step of 1 fs under the following conditions. The temperature of the system was controlled at 300 K using the Langevin thermostat^33^ with a collision frequency of 1 ps^–1^. The pressure of the system was maintained at 1 atm by applying the Berendsen barostat^34^ with a damping time of 1 ps and isothermal compressibility of 4.6 × 10^–5^ atm^–1^. The pressure of the system was controlled semi-isotropically (only along the *z*-component of the system, perpendicular to the GO sheet).^35^

For comparison, canonical simulations of the pure water system in contact with GO sheets with no aniline were also carried out with different force-fields along with the Tersoff-OPLSAA-SPCE force-field described above. The results were compared to those from ab initio MD simulations by Rolf et al. These include simulations with just the OPLS-AA representation of the sheet along with the SPC/E water model. Another set of simulations were carried out with the polarizable Drude oscillator model for the system (both the sheet and the water, specifically the SWM4-NDP water model).^36,37^ A third set of simulations used the OPLS-AA model for the GO sheets and the E3B water model for the waters.^38^ All nonpolarizable force-field simulations used the mixing rules from the OPLS-AA force-field for the water–sheet interactions. These simulations are discussed in greater detail in the Supporting Information.

The aniline molecule was built and optimized using the Avogadro^39^ application. This aniline molecule was then solvated in the aqueous phase of the equilibrated GO–water system. The approximate distance at the start between the GO sheet and the aniline molecule was approximately 45 Å. The dimensions of this aniline-containing system were approximately 25 × 25 × 105 Å^3^. The bonded and nonbonded interactions of the aniline molecule were modeled using the OPLS-AA force-field.^24^ The energy of each system with the aniline molecule was minimized. Following this, each system was equilibrated as before followed by a 20 ns production run with a 1 fs time step. At the end of this simulation, the dimensions of the system were approximately 22 × 22 × 105 Å^3^.

In order to study the effect of different salts on the adsorption of aniline on to GO surface, adsorption studies were carried out with several aqueous salt solutions with both GO sheets. For this study, two salts namely, NaCl and NaI, have been used. A concentration of 0.5 mol dm^–3^ salt solution was used, in accordance with experimental studies in the literature.^37^ In order to obtain this concentration, with the GO_4/1_ system, 16 Na^+^ ions and 16 ions of the counterions of each salt were added, and with the GO_2/1_ system, 18 Na^+^ ions and 18 of the respective counterions were added. The salt-containing systems were equilibrated under the same conditions specified for the GO–water systems, and the aniline molecule was similarly added to the salt-containing systems.

### 2.2 Umbrella Sampling Simulations

In order to capture the aniline molecule at different points in the course of its adsorption to the GO sheet, an enhanced sampling method, umbrella sampling,^40^ was used. The equilibrated systems described above were used as the starting configuration of the umbrella sampling process. The distance (in the *z*-direction) between the center of mass (COM) of the GO sheet and the COM of the aniline molecule was used as the collective variable. A total of 27 umbrella sampling windows with a step size of 1.5 Å were simulated for each system. A harmonic potential with a force constant of 3 kcal mol^–1^ was used to constrain the aniline molecule to the collective variable distance in the *z*-direction. Each umbrella sampling window was simulated for 34 ns, and all simulations were run with the same settings used in the second equilibration simulation mentioned above (in part a).

The weighted histogram analysis (WHAM) method^41,42^ was used to derive the potential mean force of the adsorption process from the umbrella sampling simulations. For this analysis, the last 30 ns of each umbrella sampling window was used. These 30 ns sections of data were divided into four equal portions with 7.5 ns of simulation time. Using the block averaging method, the standard deviation for the potential mean force curve was calculated.

### 2.3 Replica Exchange Molecular Dynamics (REMD)

REMD simulations were independently conducted with the GO_4/1_–NaCl and GO_4/1_–NaI systems in order to determine ion proximity to the GO sheet and to confirm whether the regular canonical simulations had adequate sampling.^43^ In REMD simulations, replicas of the system are simulated at different temperatures, and configurations are swapped between replicas using the Metropolis criterion. For the GO_4/1_–NaCl and GO_4/1_–NaI systems, the replica system temperatures were distributed every 5 K between 300 and 350 K and were each simulated for 10 ns. Swapping attempts were carried out every 2000 timesteps. The replica corresponding to 300 K was used for the ion proximity analysis in addition to trajectories from umbrella sampling simulations.

### 2.4 Analyses

#### 2.4.1 Proximity of Salt Ions to the GO Surface

To gain an understanding of the contribution of the salt ions to the adsorption process, the proximity of each type of ion (Na^+^, I^–^, Cl^–^) to the GO sheet was analyzed. The umbrella sampling windows with the collective variable set to 2.5, 4.0, and 14.5 Å were used for this analysis. The probability distribution of the distance (in the *z*-direction) between the center of mass of the GO sheet and the salt ion was determined.

#### 2.4.2 Orientation of the Aniline Molecule (θ_A_)

**Figure 2:**
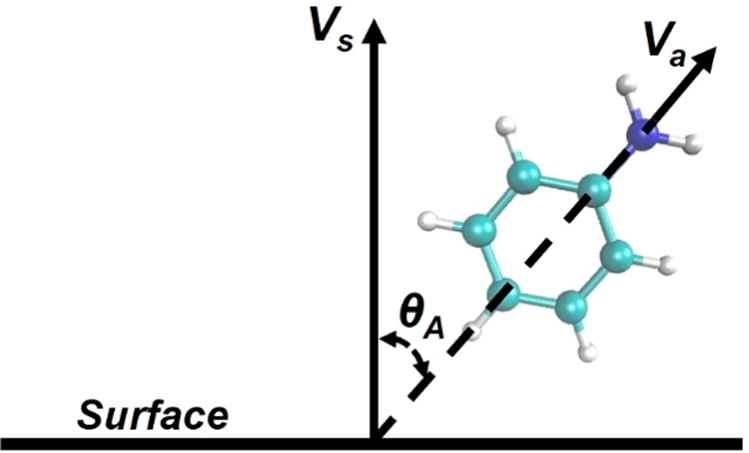
Schematic of the aniline orientational angle, θ_A_, and its defining vectors.

The orientation of the aniline molecule with respect to the GO surface was investigated over the course of the molecule’s approach to the surface. The orientation of the aniline molecule was determined by the orientational angle, θ_A_ (see Figure 2), which is defined as the angle between the vector connecting the *para* position carbon atom and the nitrogen atom of the aniline molecule (V_a_) and the normal vector to the surface of the GO sheet (V_s_). The sheet normal vector was defined in the following way: First, the carbon atom (C1) closest to the center of mass of the aniline molecule was identified. Then, the two closest carbon atoms (C2 and C3) to C1 were identified as well. Using the three carbon atoms (C1, C2, and C3), the normal vector was defined as the vector perpendicular to the plane created by the three carbons. The umbrella sampling windows with the collective variable set to 2.5, 4.0, and 14.5 Å were used for this analysis.

#### 2.4.3 Interfacial Hydration and Fluctuation of the Instantaneous Water Interface

In previous work by David et al., the instantaneous water interface of the GO–water system, based on the definition of Willard and Chandler,^44^ was used to study the effect of the level of oxidation of the GO sheet.^19^ In this work, the effect of the organic contaminant as well as the added salts on this interface are examined as a function of GO oxidation. For each frame of the simulation trajectory, the instantaneous water surface (see Supporting Information for details) was computed. The mean surface height, *z̅* was obtained by averaging the *z*-coordinates of 900 points sampled across the surface at each frame and then averaging across the entire trajectory (eq 1).
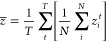
1where *N* is the total number of sample points, *T* is the total number of time steps, and *z*_*i*_^*t*^ denotes the *z*-coordinate of the *i*-th point on the surface at time step *t*. The mean surface height was used as a baseline to measure the two-dimensional, mean surface displacement of the instantaneous surface. The mean surface height on the two-dimensional surface *z̅*(*x,y*) is defined as follows:
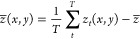
2where *z*_*t*_(*x,y*) is the height of the instantaneous water surface (centered on the *x*–*y* position of the aniline center of mass) at time *t*. The values were binned and averaged according to their 2D position relative to the aniline molecule’s center of mass to yield the surface fluctuation parameter as a function on the *xy* plane. Heatmaps of the fluctuation parameter over the coordinate grid were produced, centered on the aniline molecule’s center of mass, showing the average fluctuation of the instantaneous surface. The fluctuation parameter is defined such that a positive value corresponds to a rising of the instantaneous interface away from the GO sheet and a negative value to the surface being depressed toward the GO sheet.

The density of water as a function of distance from the instantaneous water surface was computed done previously by Pezotti et al. for the air–water and quartz–water systems and by David et al. for the GO–water system.^19,45,46^ Similar to previous work, three distinct layers were obtained based on the plot of the water density (ρ/ρ_bulk_) as a function of the distance from the instantaneous water surface (see Supporting Information for details and Figures S1 and S2). The L1 layer is the one of interest and is the region located at the GO–water interface between the GO sheet (*r* = −2 Å and ρ/ρ_bulk_ = 0) and the first minimum (*r* = 3.30 Å and ρ/ρ_bulk_ = 0.80) after the first peak (*r* = 1.40 Å and ρ/ρ_bulk_ > 1.75).^42^ As a measure of interfacial hydration, the number of water molecules near the GO–water interface, namely the L1 layer, throughout the adsorption process was obtained. The water molecules in this L1 layer were counted in each frame of the trajectory, and the average number of water molecules was obtained for each system and each umbrella sampling window.

##### 2.4.3.1 Hydrogen Bond Interactions

In order to determine the importance of hydrogen bonding interactions on the adsorption process, the change in the hydrogen bonding of the aniline molecule with the GO sheet and the water molecules as the aniline molecule gets adsorbed onto the GO surface was studied. For this analysis, five possible hydrogen bonding scenarios were identified: (1) the interaction between the nitrogen atom of the NH_2_ group of the aniline molecule and the hydrogen atoms of the water molecules, (2) the interaction between the hydrogen atom of the NH_2_ group of the aniline molecule and oxygen atoms of the water molecules, (3) the interaction between the hydrogen atom of the NH_2_ group of the aniline molecule and oxygen atoms of hydroxyl groups of the GO sheet, (4) the interaction between the hydrogen atom of the NH_2_ group of the aniline molecule and oxygen atoms of epoxy groups of the GO sheet, and (5) the interaction between the hydrogen atom of a hydroxyl group of the GO sheet and the nitrogen atoms of the aniline molecule. For this analysis, a hydrogen bond was defined when the H–O or H–N distance is less than 2.5 Å.^47^ From this analysis, the average number of hydrogen bonds of the aniline molecule was obtained as the aniline molecule gets adsorbed onto the GO surface.

## 3 Results and Discussion

Previous work by Subasinghege Don et al.^48^ on the water ordering at the GO–liquid interface showed that conventional effective pair potential based force-field representation of GO such as OPLS-AA^24^ led to overstructuring of water molecules at the GO surface when compared with the higher-level ab initio molecular dynamics (AIMD) data. From their simulations using a conventional effective two-body force-field, namely OPLS-AA,^24^ the water molecules at the interfacial regions showed a well-ordered orientation as opposed to the less-ordered orientations suggested from the more-accurate AIMD simulation data. Furthermore, David et al. showed that the AIMD simulations reproduced the key features of the experimental vibrational sum frequency generation spectra for the interfacial aqueous region near the fully oxidized and partially reduced GO surface.^19^ Hence, in order to determine a reasonably accurate force-field that reproduces the features of the AIMD simulations, the structure of the interfacial water using different force-fields including those that incorporate a many-body potential for the liquid phase as well as the GO surface was investigated. In this analysis, the orientation of the water molecules at the L1 region (interfacial water region near the GO surface) was studied with different computational models. [See Supporting Information for the details of the definitions of the L1 region (Figures S1 and S2), water orientation angles (Figures S3 and S4), and the different computational models that were used and the results of this analysis.] After comparing the results of the water orientation analysis obtained from the different computational models to the AIMD simulation results (AIMD results are taken from previous work by David et al.),^19^ the model with the Tersoff potential^22^ for the GO surface, the SPC/E water model, and OPLS-AA force-field parameters for the rest of the system was identified as the best model to accurately represent the GO–water interface. Hence, in this study, the three-body Tersoff potential^22^ was used to represent the graphene part of the GO sheets. The inclusion of this three-body potential showed a better description of the water ordering at the GO surface indicating the importance of the accurate representation of the GO sheet.

### 3.1 Free Energy Profile for Aniline Adsorption onto the GO Surface

**Figure 3:**
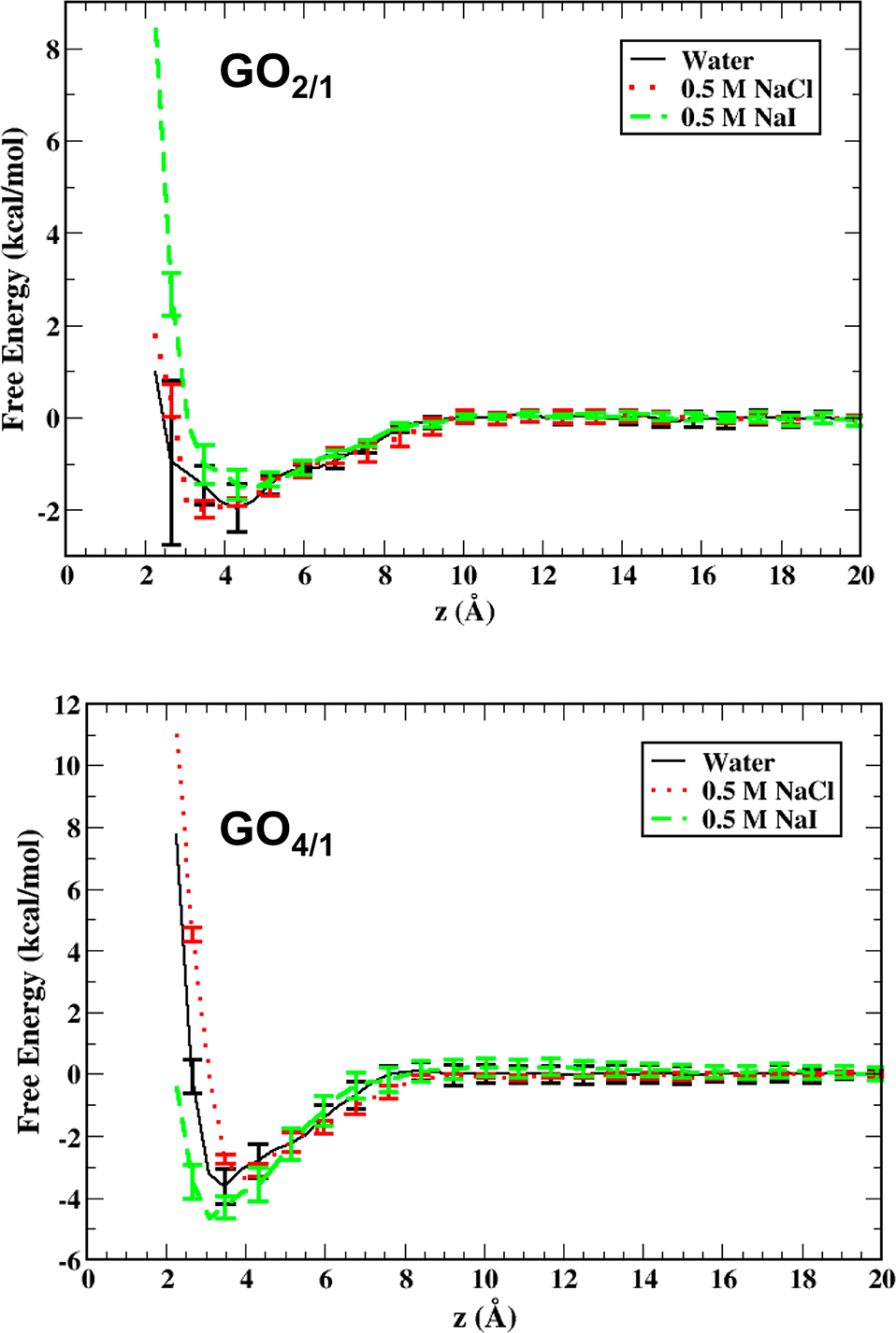
Free energy profile of the adsorption of aniline for the GO_2/1_ (top) and GO_4/1_ (bottom) surfaces in pure water and in the presence of dissolved salts as a function of the distance of the center of mass of aniline from the center of mass of the respective sheet in the *z*-direction. The solid black line is for the pure water system, the dotted red line is for 0.5 M NaCl, and the dashed green line is for 0.5 M NaI.

Enhanced sampling simulations were used to obtain the free energy profiles of the adsorption process for GO_2/1_ and GO_4/1_ at 300 K in pure water and in the presence of NaCl and NaI salts (Figure 3). The free energy profiles are shown with respect to the reference point where the aniline molecule is ∼20 Å away from the GO sheet. All of the free energy profiles exhibit no free energy barrier for adsorption and indicate GO’s ability to act as an adsorbing material. For all cases, the free energy decreases as the aniline molecule is adsorbed to the surface and then steeply increases as the aniline enters the region within the oxygen functional groups of the GO sheet.

The depth of the free energy minima reveals that the GO_4/1_ case is clearly favored over the GO_2/1_ case indicating that the adsorption of aniline is thermodynamically more favorable with the GO_4/1_ sheet. The addition of NaCl in the solution has little effect on the free energy minimum in both the GO_2/1_ and GO_4/1_ cases. On the other hand, the addition of NaI results in different effects between the two oxidation cases. In the GO_4/1_ case, the free energy minimum is decreased by the addition of NaI, making the adsorption more thermodynamically favorable compared to the system without salts. In the GO_2/1_ case, the free energy of the minimum is increased, making the adsorption less favorable compared to the GO_4/1_ case. Given the somewhat large error bars, the finer details of the free energy profiles cannot be interpreted; however, the trends remain clear. Experimental studies on the adsorption of aromatics on activated carbon with differing levels of oxygen-containing groups have also shown a similar trend.^49^ The focus of this computational study is to obtain insight into the effect of salts at the *molecular level* in the adsorption process. The solvation environment of the aniline molecule and salt ions at the GO surface can play a significant role in altering the thermodynamic favorability of the adsorption, and hence, these effects are explored in greater detail in the proceeding sections.

### 3.2 Ion Proximity

**Figure 4:**
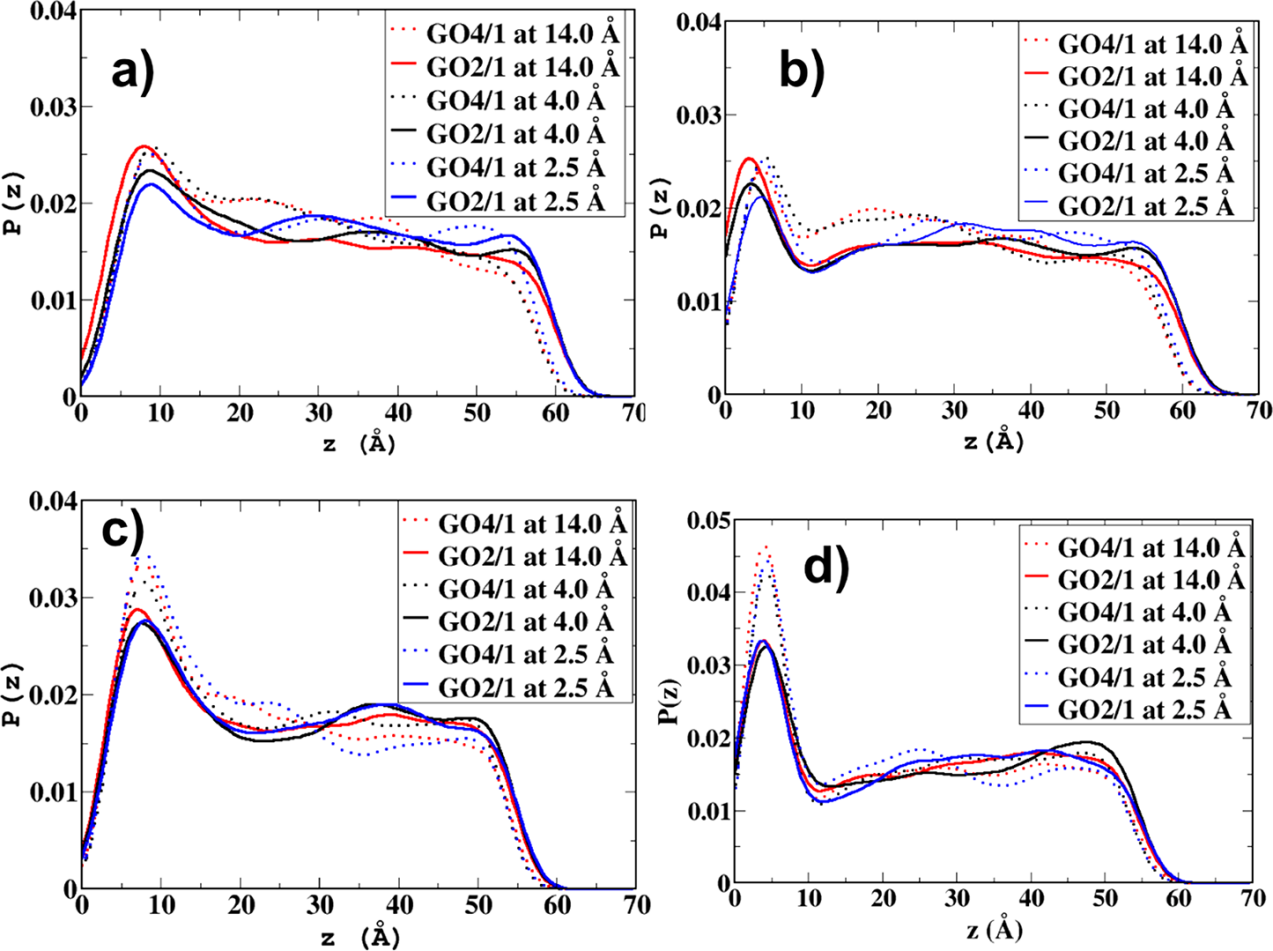
Comparison of the probability distribution of finding (a) Na^+^ for NaCl solution, (b) Cl^–^ for NaCl solution, (c) Na^+^ for NaI solution, and (d) I^–^ for NaI solution as a function of distance (*z*) of the ion in the *z*-direction from the GO center of mass for three different umbrella sampling windows, namely, when the aniline is at 2.5 Å from the center of mass of the sheet in the *z*-direction (blue), 4.0 Å (black), and 14.5 Å (red). The GO_4/1_ case is represented by a dotted line, and the GO_2/1_ is represented by solid lines. The insets show the peaks for the respective graphs. The area under each line is normalized to unity.

To further determine the influence of the added salts, the proximity of the salt ions to the GO surface was investigated. Figure 4 summarizes the probability distribution of the distance (in the *z*-direction) between each of the salt ions and the COM of the GO surface for GO_4/1_ and GO_2/1_ at 300 K from three umbrella sampling windows simulations where the aniline molecule is restrained to 2.5, 4.0, and 14.5 Å (far from the GO surface) from the GO center of mass. The probability distributions of the salt under study for GO_4/1_ for the NaI system (Figure 4c,d, dotted lines) indicate a clear preference of the ions for the interface, with the preference more pronounced for the iodide anion as compared to the sodium ion. For the NaCl case (Figure 4a,b, dotted lines), there is no clear significant interfacial preference of the ions for the GO_4/1_ sheet. In the GO_2/1_ case as well, there is a greater propensity for the ions of the NaI salt (Figure 4a,b solid lines) to be near the interface, albeit with a lower probability as compared to the GO_4/1_ case. Furthermore, the ions of the NaCl solution (Figure 4, top, red solid lines) do not show a strong preference for the GO_2/1_ case.

Of all the ions in the study, the iodide ion has the greatest affinity for the interfaces under study as compared to the sodium and chloride ions. This is reminiscent to what is seen for alkali halide salts in the air–water system, wherein the iodide ion shows the greatest propensity for the air–water interface compared to the other halides, while the alkali metal ions prefer to remain in the bulk.^50^ This can be attributed to the favorable water–water interactions as the iodide gets desolvated as it moves to the surface, which more than compensates for the decrease in ion–water energies due to desolvation, which is greatest for the large iodide ion. As the aniline molecule approaches the GO sheet, the distribution of ions at the surface is almost unaffected for the GO_4/1_–NaCl (Figure 4a,b inset, dotted lines) and GO_2/1_–NaI (Figure 4c,d inset, solid lines) cases. However, in the GO_2/1_–NaCl (Figure 4a,b inset, solid lines) cases, the presence of salt ions near the GO surface decreases slightly as the aniline molecule is brought closer to the surface. For the GO_4/1_ NaI solution case, the presence of the iodide ion decreased slightly as the aniline molecular approaches the interface (Figure 4d inset, dotted line). This suggests that for the latter two cases, the aniline molecule displaces or blocks access to regions of the GO sheet where these ions prefer to adsorb. Interestingly, the Na^+^ ion affinity goes down initially as the aniline approaches the GO_4/1_ surface in the case of NaI solution (Figure 4c inset, dotted line) but then goes up as the aniline adsorbs to the GO sheet.

Next, replica exchange MD simulations on the GO_4/1_ sheet were carried out. Figure S5 shows the potential energy overlap for the various replicas. Examination of the replica exchange simulation trajectories at 300 K reveals that the aniline is at the interface (unsurprising given the free energy profile of adsorption), and the probability distribution of the ions mirrors the results from the umbrella sampling windows when the aniline is close to the interface (see Figure S6). The trends discussed in the previous paragraph are also apparent from the results at 300 K from the replica exchange simulations

### 3.3 Aniline Orientation

**Figure 5:**
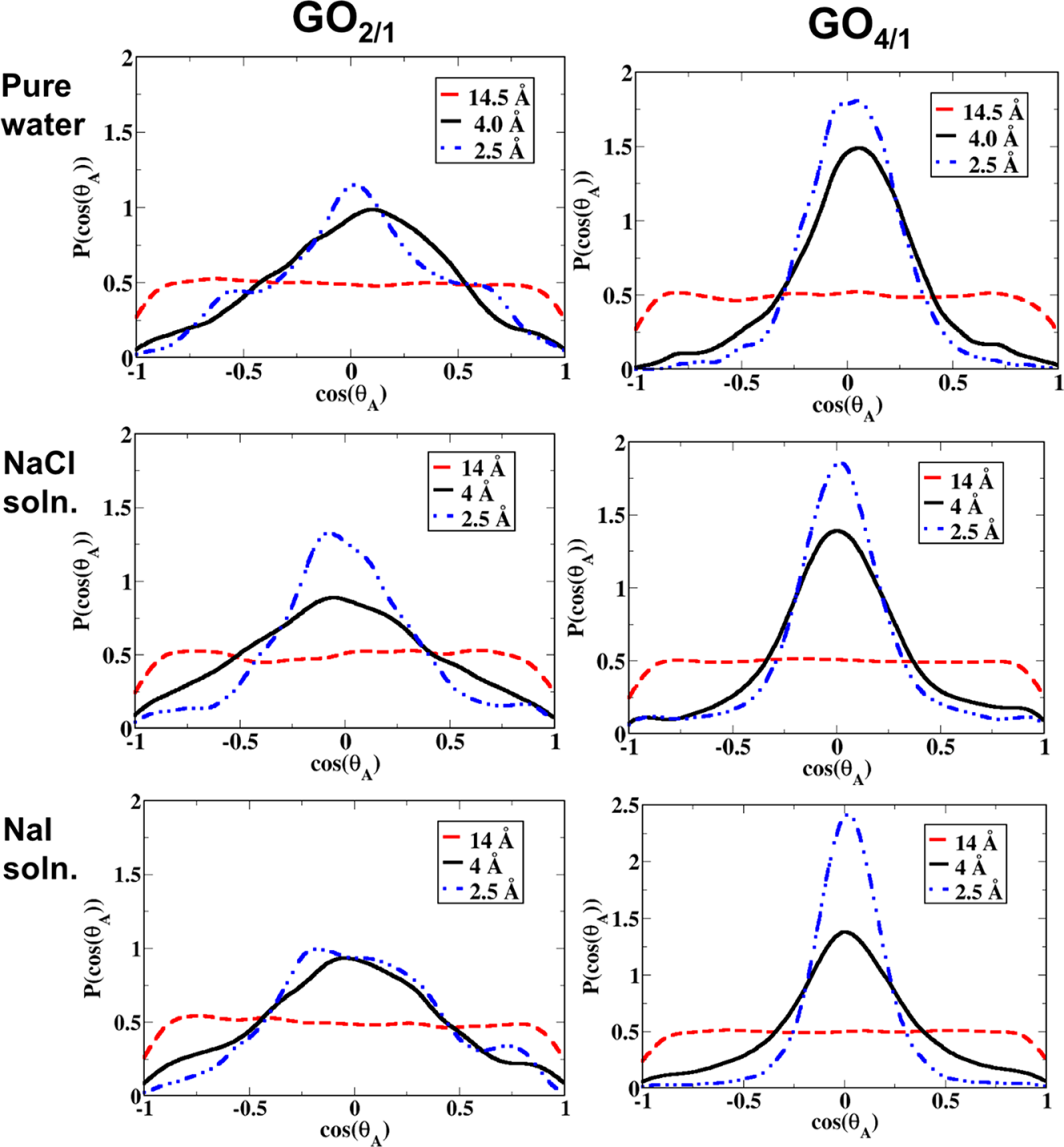
Probability distributions of the cosine of the aniline orientation angle, θ_A_, for the six systems under study from three umbrella sampling windows (4 Å in black solid line, 2.5 Å in blue dashed line, and 14 Å in red dotted line). The area under each line is normalized to unity.

The orientation of the aniline molecule as it approaches the GO surface was investigated. The angle θ_A_ was used to quantify the orientation of the aniline molecule with respect to the GO sheet. Figure 5 summarizes the probability distribution of the aniline orientation angle when the aniline is far from the sheet and when the aniline is adsorbed onto the GO sheet.

**Figure 6:**
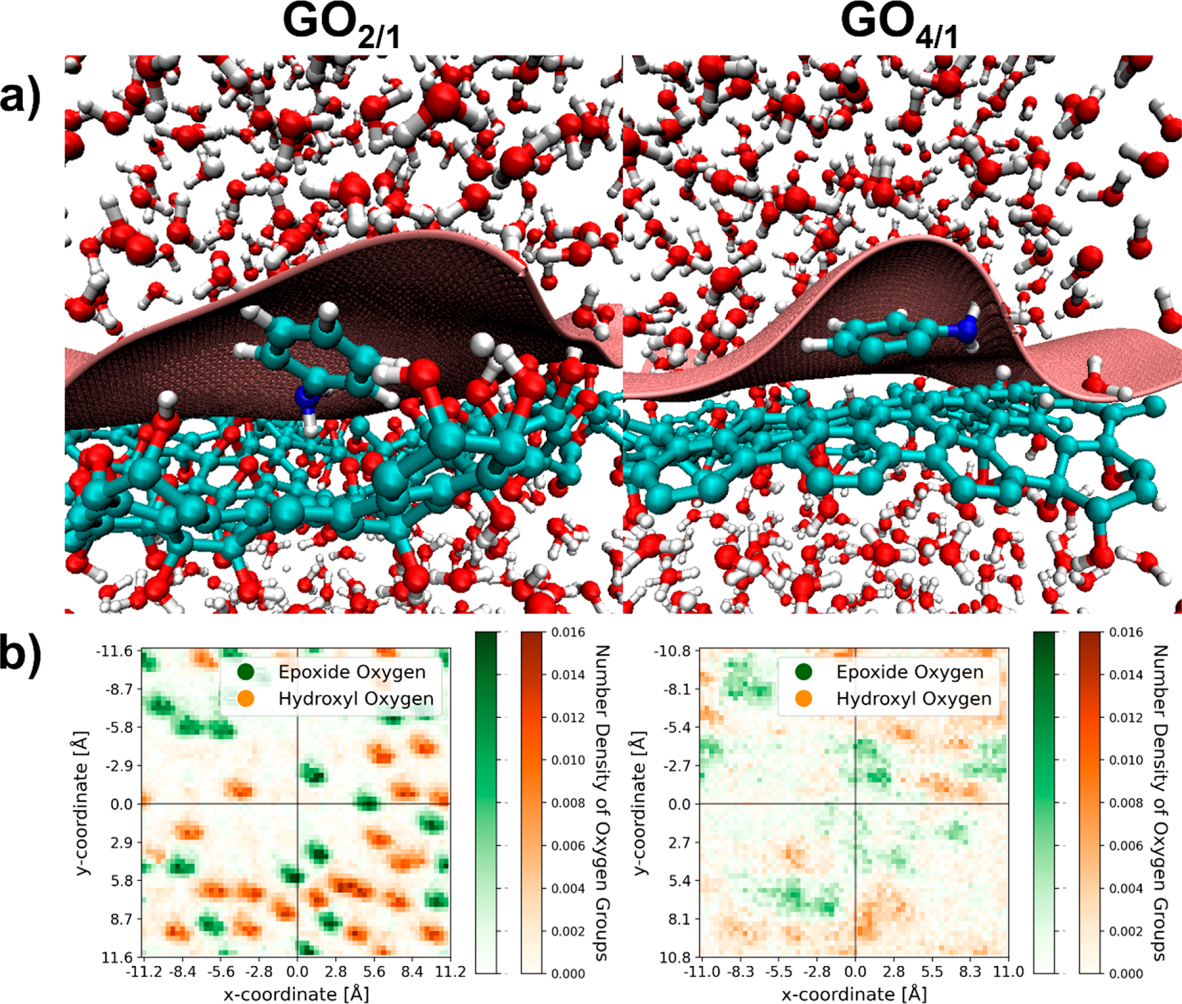
(a) Snapshot showing the orientation of aniline near the (left) GO_2/1_ surface and (right) GO_4/1_ surface from REMD simulations at 300 K with no added salt. Carbon atoms are shown in cyan, oxygen in red, hydrogen in white, nitrogen in blue, and the instantaneous water surface is shown in pink. (b) Heat maps, from the same simulations, of the distribution of oxygen groups in the *xy* plane for the (left) GO_2/1_ system and (right) GO_4/1_ system. The origin is set at the center of mass of aniline.

Examination of Figure 5 reveals that when the aniline molecule is far away from the GO surface, the distribution of the angle *θ*_A_ is, unsurprisingly, uniform with no preferred orientation toward the GO sheet, indicating weak interactions between the aniline molecule and the GO surface. However, when the aniline molecule reaches the GO–water interface (again for all three systems, namely pure water, NaCl solution, and NaI solution), a preferred orientation of the aniline molecule with an angle cos θ_A_ ≈ 0 can be seen. This angle corresponds to a configuration where the aniline molecule is parallel to the GO surface with the benzene part of the aniline molecule stacking with the GO sheet due to π–π stacking. Figure 6a shows snapshots when the aniline is close to the GO_2/1_ and GO_4/1_ surfaces. For the GO_4/1_ system, there is a sharp peak with a maximum at the cos θ_A_ ≈ 0 orientation when the aniline molecule is close to the GO surface, indicating strong π–π interactions, enabling the aniline molecule to get adsorbed more strongly, in agreement with the free energy of adsorption. For the case of GO_4/1_ with added NaI salt, the peak becomes even sharper when the aniline is adsorbed onto the GO sheet, suggesting stronger π–π stacking. On the other hand, in the case of GO_2/1_, the probability distribution of the angle is broader, although there is still a maximum at cos θ_A_ ≈ 0. The added salt makes the distribution even broader. In GO_2/1_, the higher oxidation level limits the π–π interactions, resulting in a broader distribution of angles and thus in a smaller change in the free energy of adsorption. This is also clear from the snapshot for GO_2/1_ in Figure 6, wherein the aniline is tilted with respect to the sheet. In addition, from the snapshots as well as the heat map of the distribution, centered on the center of mass of aniline, of the oxygenated groups in Figure 6b, it is clear that for the GO_4/1_ case, the aniline prefers to adsorb at the intersection of the graphene-like and oxygen-rich regions. This enables the aromatic group of aniline to π–π stack with the graphene-like region of the sheet, while the −NH_2_ group can hydrogen bond with the oxygenated groups of the sheet. For the GO_2/1_ case, the fully oxidized sheet does not have clear graphene-like regions for π–π stacking.

### 3.4 Interfacial Hydration, Hydrogen Bonding of the Aniline Molecule, and Fluctuations of the Instantaneous Water Surface

**Figure 7:**
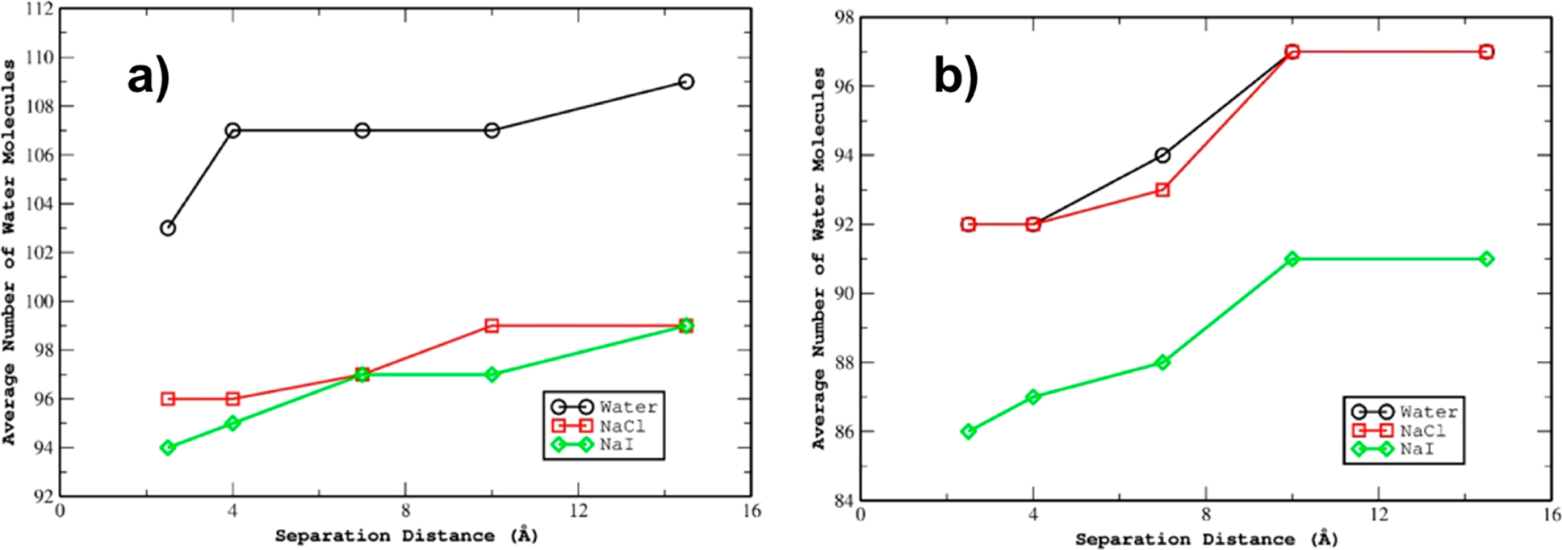
Comparison of the average number of water molecules in the L1 layer for each system as a function of distance of the aniline center of mass from the center of mass of the sheet (in the *z*-direction) for the (a) GO_2/1_ and (b) GO_4/1_ sheets.

The change in the number of water molecules in the L1 interfacial layer was analyzed over the course of the adsorption process. Figure 7 summarizes the data obtained for this analysis for the GO_2/1_ and GO_4/1_ sheets at 300 K. In the GO_4/1_ case, the water count gradually decreases (∼5 water molecules) as the aniline molecule approaches the GO surface. However, the number of water molecules that are closer to the GO surface is slightly lower in the case with NaI salt compared to the case with NaCl salt and the case without any salt. This can be correlated to the observation in Figure 4, in which the salt ions in the NaI case (for the GO_4/1_ at 300 K) are strongly coordinated to the GO surface compared to the NaCl case. Hence, slightly fewer water molecules are closer to the GO surface in the NaI system, since the Na^+^ and I^–^ ions stay in close proximity to the GO surface. In the GO_2/1_ case, in general, there are a larger number of water molecules near the interface as compared to the corresponding GO_4/1_ set of systems. As in the GO_4/1_ case, the presence of added salts decreases the number of water molecules on average at the interface. However, unlike in the GO_4/1_ case, the presence of added salt (whether NaCl or NaI) results in a very slight decrease in the number of water molecules as the aniline approaches the surface. For the pure water in contact with GO_2/1_, the decrease is much more dramatic than the salt solutions.

**Figure 8:**
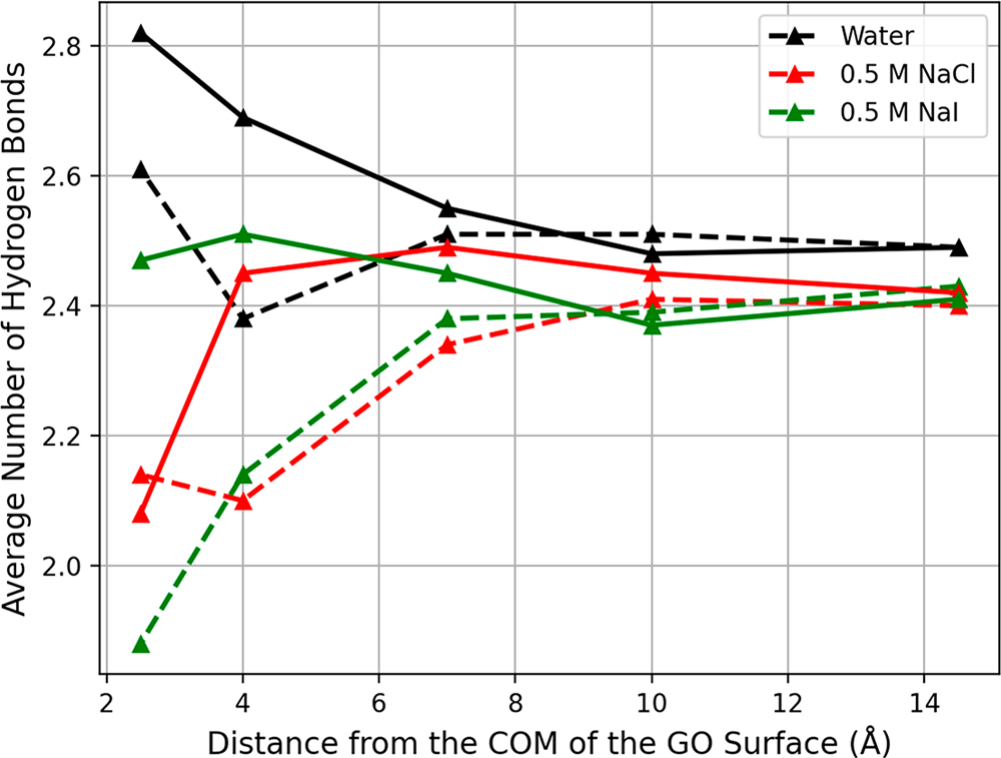
Average number of hydrogen bonds of the aniline molecule at different separation distances with respect to the GO_2/1_ (solid) and GO_4/1_ (dashed) surfaces. The pure water system is in black, NaCl solution in red, and NaI solution in green.

In order to investigate the importance of hydrogen bonding on the adsorption process of the aniline molecule in the different systems under study, the average number of hydrogen bonds that the aniline molecule forms as it gets adsorbed onto the GO surface was analyzed. Different types of hydrogen bonds that the aniline molecule can form are defined in the Methods section. This analysis was performed with the GO_4/1_ and GO_2/1_ systems as a function of distance (in the *z*-direction) between the aniline center of mass and the center of mass of the GO sheet. The results are shown in Figure 8. When comparing the data obtained, the GO_4/1_ system shows a lower average number of hydrogen bonds compared to the corresponding GO_2/1_ system when the aniline approaches the GO surface. From the analyses performed in the presence of the salt ions as well as the analyses of the fluctuations of the interfacial water molecules, it is seen that in the GO_4/1_–NaI system at 300 K the ions stay close to the GO surface, leading to a smaller number of interfacial water molecules compared to the GO_2/1_ system. In the GO_2/1_ case, the surface is completely covered with oxygen functional groups as compared to the GO_4/1_ system, and hence, the aniline molecule can form a larger number of hydrogen bonds leading to a higher average number of hydrogen bonds in general.

Table 1 shows the average number of hydrogen bonds (when the aniline is close to the sheet, specifically the 4 Å umbrella sampling window) that the aniline forms with the O atom of the hydroxyl group (A_D_–Hyd_A_), the O atom of the epoxy group (A_D_–Epo_A_), and the H atom of the hydroxyl group (A_A_–Hyd_D_). The subscript A refers to acceptor N or O atoms and the subscript D to donor H atoms to the hydrogen bond. From Table 1, it is clear that in the GO_2/1_ case that the aniline forms, on average, more hydrogen bonds with the sheet as compared to the GO_4/1_ case. In both the GO_2/1_ and GO_4/1_ cases, the dominant hydrogen bond with the sheet is with the epoxy group (A_D_–Epo_A_). The presence of added salt does not have a significant impact on the A–Epo_A_ hydrogen bonds in the GO_4/1_ case. However, in the GO_2/1_ case, there is a non-negligible decrease of the A_D_–Epo_A_ hydrogen bonds in the presence of added NaI salt, thereby contributing to the reduced propensity of aniline for the GO_2/1_ surface in this case.

**Table 1:** Average Number of Hydrogen Bonds^a^ That the Aniline Makes with the Hydroxyl and Epoxy Groups of GO_2/1_ and GO_4/1_^b^

GO_2/1_	water	+NaCl	+NaI
A_D_–Hyd_A_	0.2	0.1	0.1
A_D_–Epo_A_	0.6	0.6	0.3
A_A_–Hyd_D_	0.1	0.0	0.1

GO_4/1_	water	+NaCl	+NaI
A_D_–Hyd_A_	0.1	0.2	0.1
A_D_–Epo_A_	0.3	0.2	0.2
A_A_–Hyd_D_	0.1	0.0	0.0

a From the 4 Å umbrella sampling window.

b A_D_–Hyd_A_ refers to aniline with O of the hydroxyl group, A_D_–Epo_A_ refers to aniline with O of the epoxy, and A_A_–Hyd_D_ refers to aniline with H of the hydroxyl group of the sheet.

### 3.5 Fluctuations of the Instantaneous Water Surface

**Figure 9:**
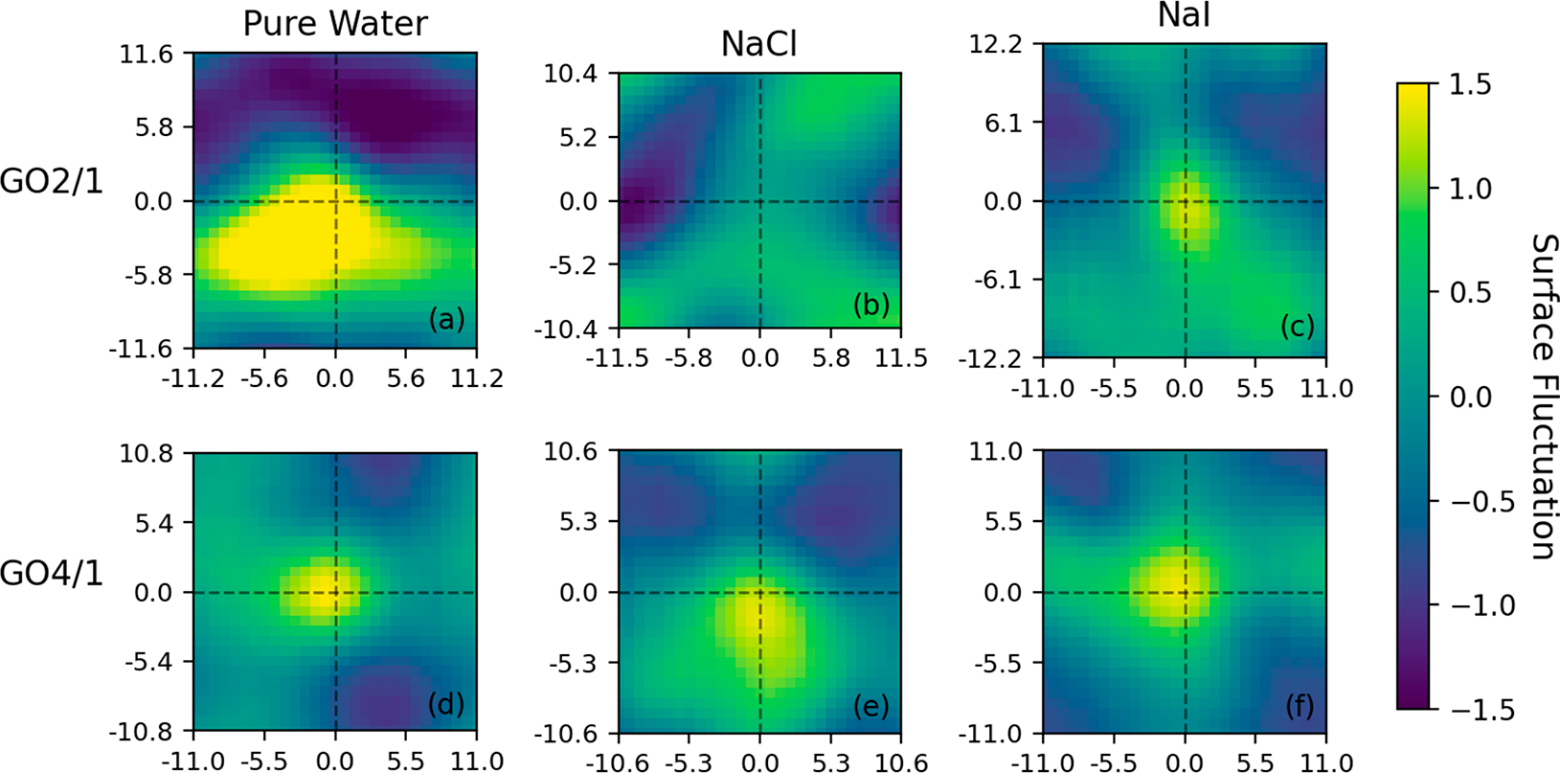
2D heat map of the average fluctuations in the height of the instantaneous surface (from the umbrella sampling window set to 4.0 Å) on the *xy* plane. Histograms are centered on the aniline center of mass.

Fluctuations of the instantaneous interface were investigated through heatmaps of the surface fluctuation parameter on the *xy* plane, parallel to the GO sheet. The heatmaps were centered on the aniline molecule’s center of mass (COM) and are presented in Figure 9 for the umbrella sampling window set to 4.0 Å. A positive fluctuation denotes a rising of the instantaneous surface, away from the GO sheet, and a negative fluctuation corresponds to a depression of the instantaneous surface, toward the GO sheet. The fluctuations of the instantaneous surface are, thus, a measure of the movement of water density along the GO–water interface due to the aniline molecule, revealing how water molecules near the interface are being displaced by the aniline molecule. For comparison, Figure S7 in the Supporting Information shows the average fluctuations of the instantaneous surface when the aniline is far from the GO_2/1_ and GO_4/1_ surfaces.

In the GO_4/1_ case, a strong positive peak is observed centered on the aniline center of mass, indicating the displacement of water molecules between the aniline molecule and the GO sheet. The presence of added salts does not have a pronounced effect on the fluctuations of the instantaneous water surface. The analysis of the orientation of the aniline molecule near the GO_4/1_ surface, in previous sections, shows a strong preference for the parallel stacking of the aniline with the graphene-like region of the GO_4/1_ irrespective of the presence of added salts. In the GO_2/1_ case with no added salt, large fluctuations are observed, which is in keeping with the broader distribution of orientations of the aniline molecule combined with a larger number of interfacial waters and hydrogen bonds of the aniline. The addition of salt ions suppresses these fluctuations in the GO_2/1_ case, decreasing the large positive and negative regions of fluctuation. This is not surprising, given the smaller number of interfacial water molecules and the lower number of hydrogen bonds of the aniline, in the presence of added salt.

The above results give rise to a picture of a GO_2/1_ surface that is hydrophilic, both due to the large number of oxygenated groups as well as the presence of more interfacial water as compared to the GO_4/1_ case. The aniline forms more hydrogen bonds when it adsorbs on the GO_2/1_ surface as compared to the more-reduced form. However, the strong π–π interactions between the aromatic ring of the aniline and the graphene-like region of the sheet in the more-reduced form (GO_4/1_), as evident from the aniline orientational analysis, give rise to the greater affinity of the aromatic compound to GO_4/1_. The presence of iodide ions at the interface increases the orientational ordering of the aniline in the GO_4/1_ case, further stabilizing the aniline adsorption. Since chloride ions are not seen preferentially near the GO_4/1_ surface, it is not surprising that the addition of NaCl does not greatly affect the aniline ordering and the free energy minimum, as compared to the more-oxidized case.

## 4 Conclusions

The use of graphene oxide in removing organic toxic waste materials from solution is gaining momentum. However, a detailed molecular perspective of the interfacial region where the adsorption takes place is lacking. Here, the adsorption of an aromatic compound, namely aniline, onto the graphene oxide surface was investigated as a function of the oxidation level of the GO sheet and in the presence of added salts using molecular simulations. The force-field that was used to simulate the system was first validated against ab initio MD results. Interestingly, the force-field that allowed for the flexibility of the graphene oxide sheet, namely the Tersoff potential, was found to give the most reasonable representation of the graphene oxide–water interface. The computational investigations of aniline adsorption on the GO sheet revealed the importance of the oxidation level of the GO surface as well as the effect of added sodium halide salts. The degree of oxidation changes the interfacial water environment, with the more-oxidized hydrophilic form essentially presenting a water-rich region that can solvate interfacial ions. The more-reduced form is more hydrophobic but, more importantly, has distinct aromatic and hydrophilic domains. The presence of distinct graphene-like regions and hydrophilic oxygenated regions of the more-reduced form, GO_4/1_, has a significant impact on the adsorption of aromatic contaminants like aniline from aqueous solutions. The free energy minimum for bringing aniline to the GO_4/1_ for the pure water case is lower than the corresponding GO_2/1_ system. The addition of sodium iodide salt lowers the free energy minimum further when the aniline adsorbs to the GO_4/1_ surface but raises it for the GO_2/1_ case. NaCl, on the other hand, does not have much of an effect on the free energy profile. In the former case, iodide ions have an affinity for the interface, whereas in the latter the chloride ion shows no clear marked affinity for the interface.

## Supporting Information Available

Additional details and comparison of different water models including interfacial water density and orientation analysis for GO_2/1_ and GO_4/1_, REMD potential energy distribution for different replicas and REMD results at 300 K for ion distribution, fluctuations of the water instantaneous surface when aniline is far from the GO surface (PDF)
