# Supplementary material for: Adsorption Studies at the Graphene Oxide–Liquid Interface: A Molecular Dynamics Study
Source: J Phys Chem C Nanomater Interfaces. 2023 Mar 20;127(12):5920–30. doi: 10.1021/acs.jpcc.2c07080 (PMC10069394; doi:10.1021/acs.jpcc.2c07080)
Supplement: Supplementary file 1 — jp2c07080_si_001.pdf [file jp2c07080_si_001.pdf]

Supporting Information

Adsorption Studies at the Graphene Oxide – Liquid Interface: A Molecular Dynamics Study

Visal Subasinghege Don,[a] Lukas Kim,[a,b] Rolf David,[a,c] Julia A. Nauman,[a,d] and Revati Kumar[a]*

[a]Department of Chemistry, Louisiana State University, Baton Rouge, Louisiana 70803-1804, United States.

[b] Current affiliation: Department of Chemistry, University of California Berkeley, California 94720-1462

[c] Current affiliation: PASTEUR, Department of Chemistry, École Normale Supérieure, PSL University, Sorbonne Université, CNRS, 75005 Paris, France

[d] Current affiliation: Department of Chemistry, Massachusetts Institute of Technology, Cambridge, Massachusetts 02139 - 4307, United States

Comparison of Different Computational Models

The computational model that should be used to model the adsorption process at the GO-liquid interface should represent the governing interactions at the interfacial regions in an accurate manner. In order to determine the best computational model to be used to model the relatively long adsorption simulations, the orientation of the water molecules in the L1 region was determined with different computational models and compared with the results from the more accurate, yet computationally expensive AIMD simulation data. The AIMD simulations are from the work of Rolf et al.[1] The different computational models that were examined and the comparisons of the water orientations with the AIMD data (Figure S3 and S4) are as follows.

(a) AIMD (revPBE functional, and the empirical D3 dispersion, with a DZVP-MOLOPT-SR basis set and GTH pseudopotentials). These trajectories were taken from the work by David et al. [1]

(b) Classical MD (OPLS-AA force field and SPC/E water model) [2-3]

(c) Classical MD (Drude-oscillator model and polarizable SWM4-NDP water model) [4-5]

(d) Classical MD (OPLS-AA force field and E3B water model) [2, 6]

(e) Classical MD (Tersoff[7-8]potential on the graphene part of the GO surface, OPLS-AA force field, and SPC/E water model).

All simulations were carried out on the same systems that were used in the AIMD simulations of David et al. A brief description is provided below. The GO sheets are the same as in the current work. The sheets were minimized and a short NPT run was carried out after which 265 water molecules were added on one side of the GO sheet generating a water layer of a thickness of 20 Å, above which a 70 Å layer of vacuum was introduced. This resulted in a box with a z dimension of 104 Å. All simulations were carried out in the NVT ensemble at 300 K with periodic boundary conditions and with the PPPM based Ewald method for the electrostatic interactions.

The Average Fluctuations of the Water Density and Water Layers

In order to differentiate various types/layers of water in the system, a definition of the GO-water interface was required. Therefore, to characterize the GO-water interface, the Willard-Chandler instantaneous interface was used. [9] In this method Gaussian functions are added at each point where an oxygen atom of a water molecule is located to create a continuous coarse-grained density distribution. The instantaneous interface is the surface corresponding to half the bulk density. Figure S1 shows the representation of the $GO_{2/1}$ sheet used in these GO-water studies with the instantaneous water interface. Moreover, to define the different types of water layers with respect to the instantaneous interface, the ratio of the average water density to the bulk density of water as a function of the distance to the instantaneous interface was calculated. Through this analysis, well-defined water layers, based on the minima in the density distributions were identified. The studies conducted in the past on interfacial aqueous systems have also seen this kind of well-defined layering. [1, 10-11] The structural analysis performed to study the ordering of different water molecules in the system were conducted on these interfacial layers.

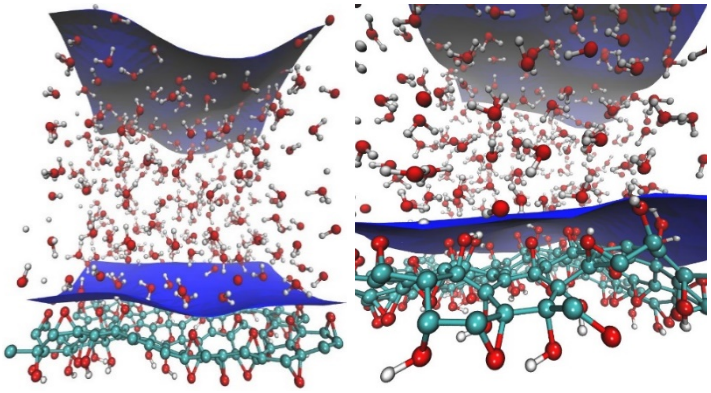

Figure S1. Snapshot of the instantaneous interface in the $GO_{2/1}$-water system (on the left) and a zoomed-in view of the instantaneous interface closest to the GO sheet (on the right). The two instantaneous interfaces of the system are represented in blue color. The atoms of carbon are represented in cyan, oxygen in red, and hydrogen in white.

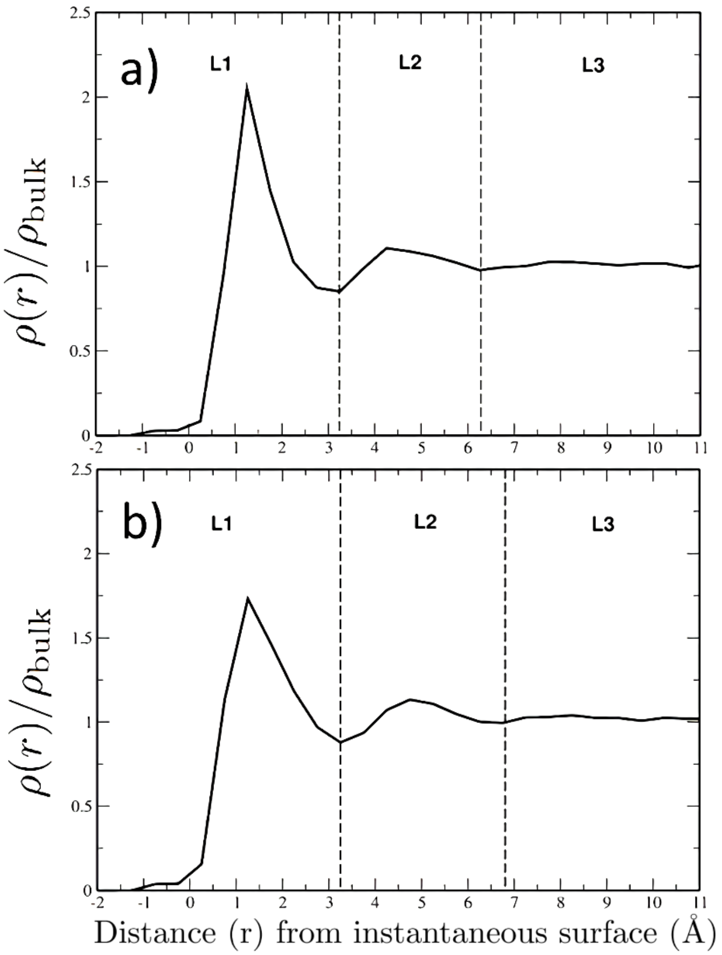

Figure S2. The mean water density as a function to the distance from the instantaneous water surface from simulations (a) $GO_{4/1}$, and (b) $GO_{2/1}$ systems in contact with water. The regions L1, L2 and L3 are marked.

Figure S2 shows the ratio of the density of water over the bulk density of water as a function of the distance to the instantaneous water interface for the two GO surfaces used in the study simulated using Tersoff-OPLSAA force-field. From this figure well-defined layers of water can be distinguished, namely, L1, L2, and L3 layers as the distance from the instantaneous interface increases. For the comparison of the water orientation with

the different computational models used to determine the accurate method to conduct the adsorption studies, the water molecules of the L1 region were used.

Interfacial Water Orientation Analyses

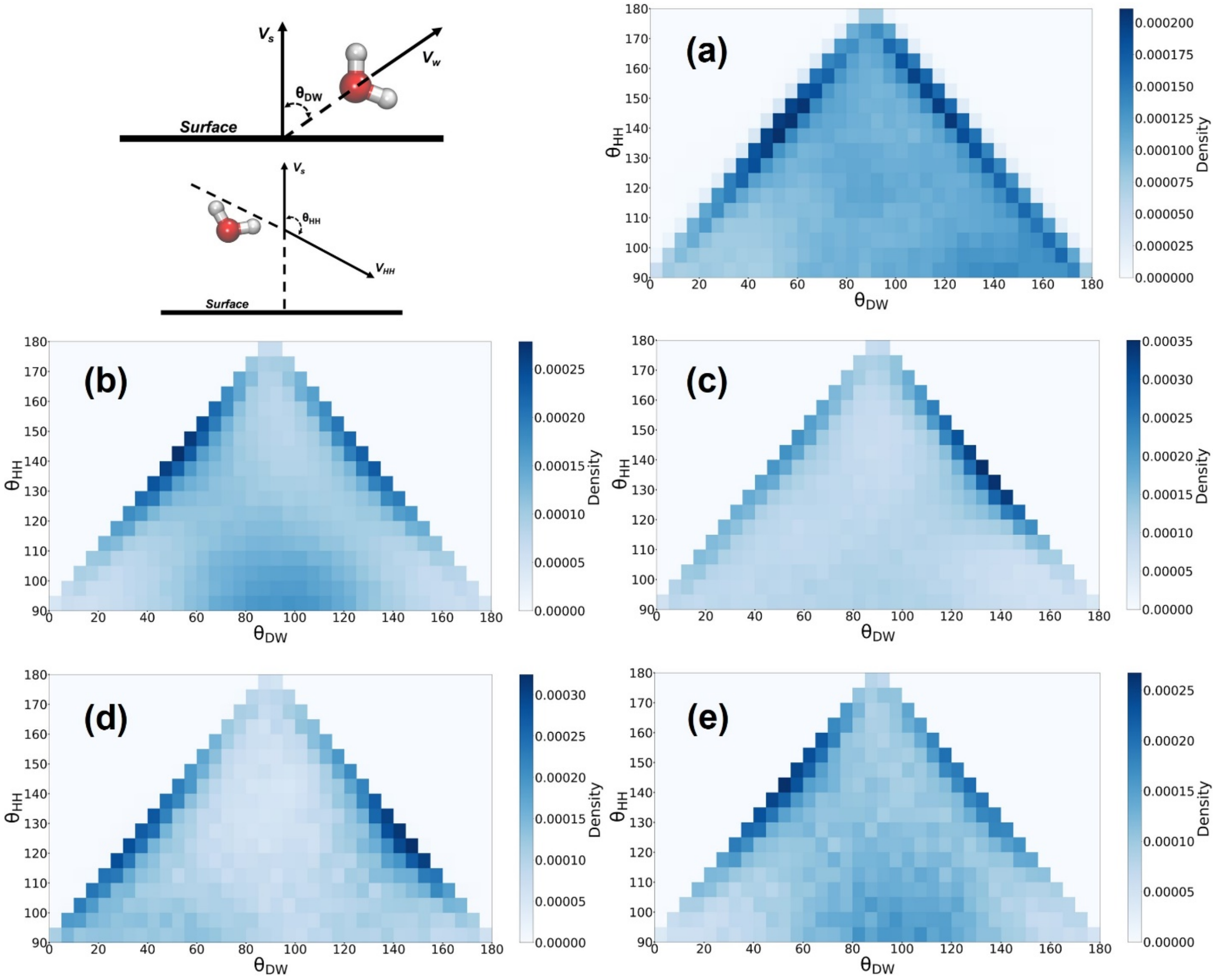

S3. Definition of the two types of water orientation angles, and two-dimensional (2D) histograms of the joint distribution of the θ_{DW} and θ_{HH} angles of L1 layer for different computational models (a) AIMD, (b) OPLSAA - SPC/E , (c) Drude - SWM4-NDP, (d) OPLSAA - E3B and (e) Tersoff-SPCE mentioned above for the **$GO_{2/1}$** system

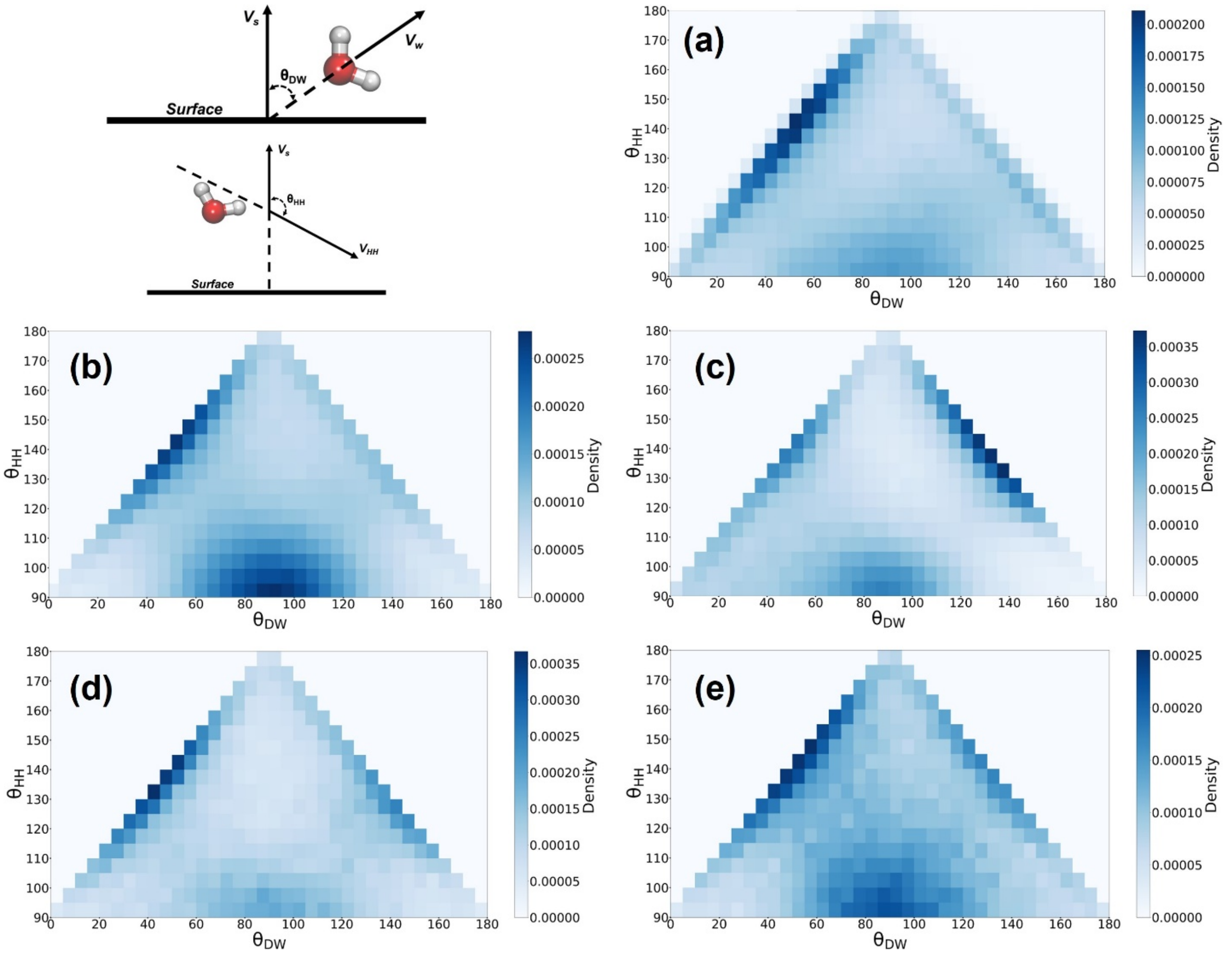

S4. Definition of the two types of water orientation angles, and two-dimensional (2D) histograms of the joint distribution of the θ_{DW} and θ_{HH} angles of L1 layer for different computational models (a) AIMD, (b) OPLSAA - SPC/E , (c) Drude - SWM4-NDP, (d) OPLSAA - E3B and (e) Tersoff-SPCE mentioned above for the **$GO_{4/1}$** system

$GO_{2/1}$

The L1 layer of the AIMD simulation for the $GO_{2/1}$ case shows two main configurations. The first orientation consists of values ranging from 50° to 55° and from 130° to 150° for θ_{DW} and θ_{HH} respectively. This configuration corresponds to an orientation with one hydrogen atom of the water molecule pointing away from the instantaneous interface. The second orientation is with values ranging from 110° to 130° and from 160° to 140° for θ_{DW} and θ_{HH} respectively with one hydrogen atom of the water molecule pointing towards the instantaneous interface. Besides these two main orientations, **the L1 layer shows a diffuse angle distribution. This shows that there can be several probable orientations of the water molecule indicating a less ordered water structuring at the L1 layer**. Comparing the L1 regions of the other simulations to the L1 region of the AIMD simulation, most of them fail to capture the broad angle distribution of the water molecules. For example, the simulation performed with the OPLS-AA force field and the SPC/E water model shows a structured interface with three distinct probable orientations of the water molecules with. The simulation with the Drude oscillator model and the SWM4-NDP water[198] model (Figure 3.6A(c)L1) shows only two main orientations of the water molecules once again indicating a well-structured arrangement of the water molecules in the L1 layer, which is in contrast with the AIMD data. On the other hand, the simulation performed with the OPLS-AA[35] force field with the E3B water model shows a slightly broader distribution of the water orientation with two major orientations. The simulations performed with the OPLS-AA[35] force field with the Tersoff potential and the SPC/E water model give rise to L1 waters that are less structured (though not as much

as the AIMD) in better agreement with the distribution of the L1 layer of the AIMD simulation with broader distributions.

$GO_{4/1}$

The AIMD shows a distinctly strong population for region (a) ranging from 40° to 80° and from 120° to 160° for θ_{DW} and θ_{HH} respectively. Region (b) has a smaller population for 70° to 110° and from 90° to 110° for θ_{DW} and θ_{HH} and an even smaller one for region (c) ranging from 120 to 150 and 130 to 110 for θ_{DW} and θ_{HH} respectively. Furthermore, the distribution is more diffuse in general for the AIMD compared to the force-field simulations. The OPLS-AA/SPCE force-fields gives rise to a very strong population for region (b). The Drude oscillator simulations give a very strong distribution for region (c). The OPLS-AA/E3B gives a much smaller region (b) distribution. The Tersoff simulations, while no means perfect has the right ordering with region (a) as the highest, followed by (b) and then (c) (although (c) is more populated than the AIMD case).

Hence, from the comparisons of the 2D histograms of the joint distribution of the θ_{DW} and θ_{HH} angles of L1 layer for the different computational models with the AIMD simulation data, the model (e) where Tersoff potential was used on the graphene part of the GO surface showed the best overall representation of the wide angle distribution for both sheets.

Replica Exchange MD

Acceptance ratios were approximately 20% for all temperatures for both systems.

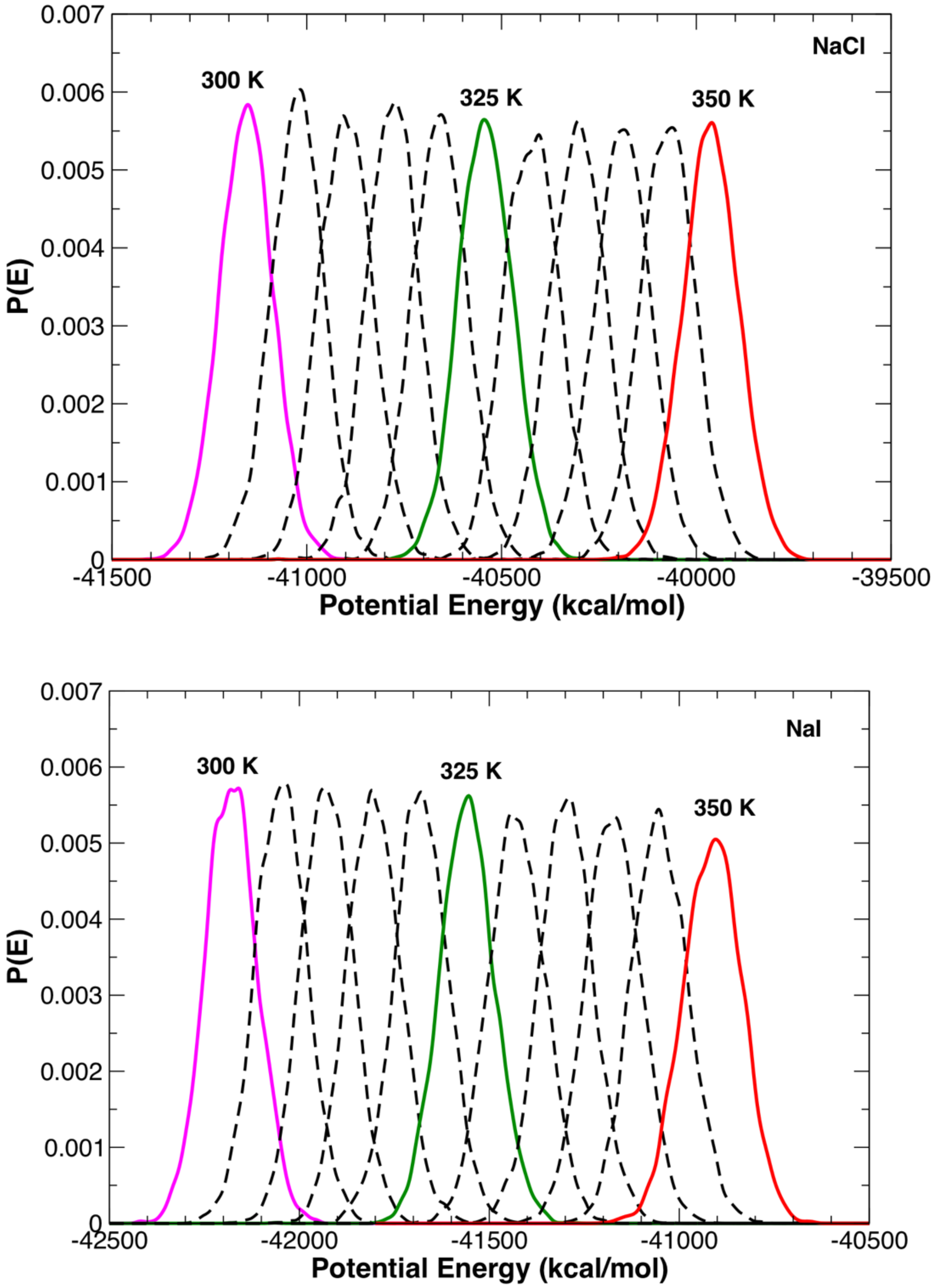

Figure S5: The potential energy distribution for the different replicas $GO_{4/1}$ -NaCl and $GO_{4/1}$ - NaI. The Kernel Density approximation was used to smoothen the curves

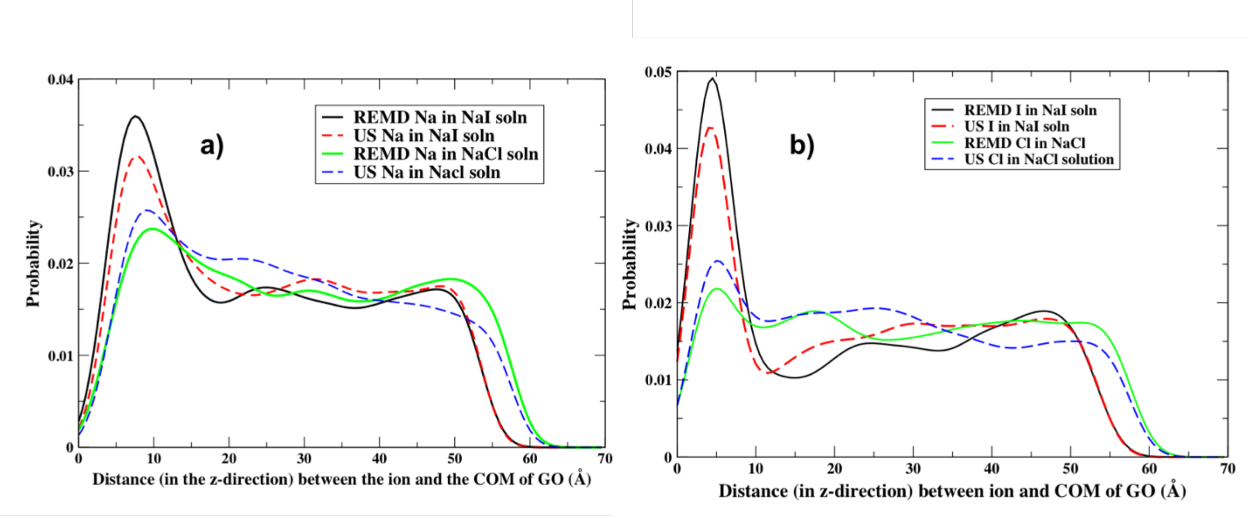

Figure S6. Probability distribution of the distance in the z-direction between the COM of the $GO_{4/1}$ sheet an the salt ions for (a) Na^+ in NaI and NaCl and b) I- and Cl- from REMD simulations at 300 K and Umbrel Sampling simulation window at 2.5 Å.

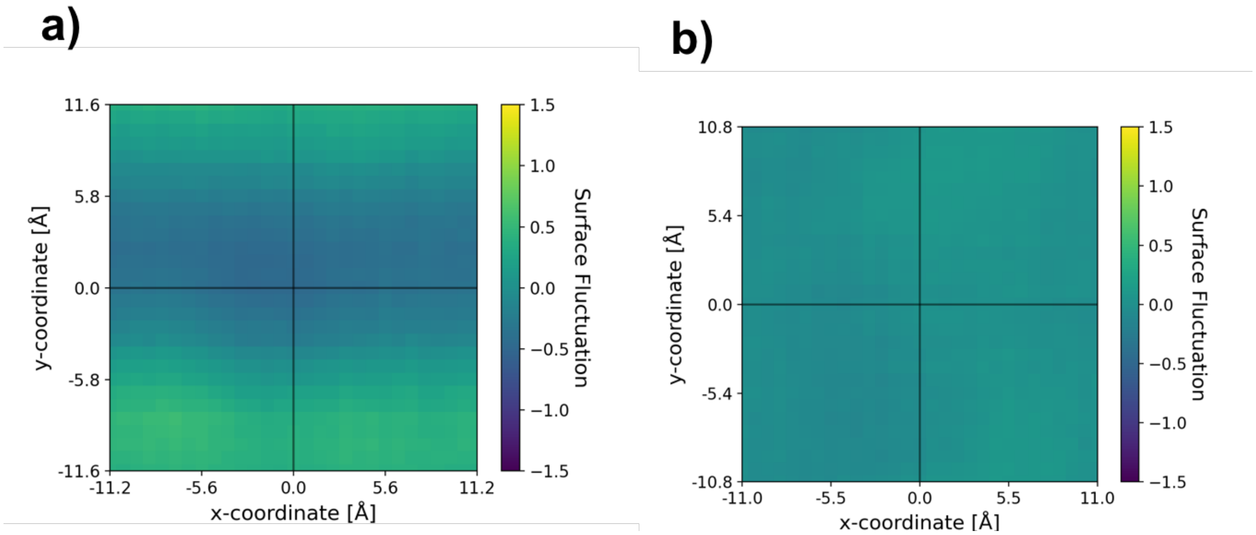

Figure S7. 2D Heat map of the average fluctuations in the height of the instantaneous surface (from the umbrella sampling window set to 25.0 Å) on the xy plane for a) $GO_{2/1}$ and b) $GO_{4/1}$. Histograms are centered on the aniline center of mass.

LAMMPS Input Files and Tersoff Parameters

# With no ions (Pure water simulation)

#Atom types

**1**	**Water O**
**2**	**Water H**
**3**	**GO C**
**4**	**Epoxy O**
**5**	**Hydroxyl O**
**6**	**Hydroxyl H**
**7**	**Aniline C1**
**8**	**Aniline C2**
**9**	**Aniline H1**
**10**	**Aniline N**
**11**	**Aniline H2**

```
boundary     p p p
units        real
atom_style   full

bond_style harmonic
angle_style harmonic
angle_style harmonic
dihedral_style opls
improper_style harmonic

# geometry
read_data        min.data

# potentials
pair_style hybrid lj/cut/coul/long 10.0 10.0  tersoff
pair_coeff   * * tersoff C.tersoff NULL NULL C NULL NULL NULL NULL NULL NULL NULL NULL
pair_coeff   1 1 lj/cut/coul/long 0.1554 3.16557
pair_coeff   1 2 lj/cut/coul/long 0.0 0.0
pair_coeff   1 3 lj/cut/coul/long 0.0930 3.2807
pair_coeff   1 4 lj/cut/coul/long 0.1475 3.0299
pair_coeff   1 5 lj/cut/coul/long 0.1626 3.1174
pair_coeff   1 6 lj/cut/coul/long 0.0 0.0
pair_coeff   1 7 lj/cut/coul/long 0.1087 3.3523
pair_coeff   1 8 lj/cut/coul/long 0.1043 3.3523
pair_coeff   1 9 lj/cut/coul/long 0.0683 2.7678
pair_coeff   1 10 lj/cut/coul/long 0.1625 3.2321
pair_coeff   1 11 lj/cut/coul/long 0.0 0.0
pair_coeff   2 2 lj/cut/coul/long 0.0 0.0
```

```
pair_coeff   2 3 lj/cut/coul/long 0.0 0.0
pair_coeff   2 4 lj/cut/coul/long 0.0 0.0
pair_coeff   2 5 lj/cut/coul/long 0.0 0.0
pair_coeff   2 6 lj/cut/coul/long 0.0 0.0
pair_coeff   2 7 lj/cut/coul/long 0.0 0.0
pair_coeff   2 8 lj/cut/coul/long 0.0 0.0
pair_coeff   2 9 lj/cut/coul/long 0.0 0.0
pair_coeff   2 10 lj/cut/coul/long 0.0 0.0
pair_coeff   2 11 lj/cut/coul/long 0.0 0.0
pair_coeff   3 4 lj/cut/coul/long 0.0883 3.1401
pair_coeff   3 5 lj/cut/coul/long 0.0973 3.2308
pair_coeff   3 6 lj/cut/coul/long 0.0 0.0
pair_coeff   3 7 lj/cut/coul/long 0.0651 3.4742
pair_coeff   3 8 lj/cut/coul/long 0.0624 3.4742
pair_coeff   3 9 lj/cut/coul/long 0.0409 2.8684
pair_coeff   3 10 lj/cut/coul/long 0.0973 3.3496
pair_coeff   3 11 lj/cut/coul/long 0.0 0.0
pair_coeff   4 4 lj/cut/coul/long 0.1400 2.9000
pair_coeff   4 5 lj/cut/coul/long 0.1543 2.9838
pair_coeff   4 6 lj/cut/coul/long 0.0 0.0
pair_coeff   4 7 lj/cut/coul/long 0.1032 3.2086
pair_coeff   4 8 lj/cut/coul/long 0.0990 3.2086
pair_coeff   4 9 lj/cut/coul/long 0.0648 2.6492
pair_coeff   4 10 lj/cut/coul/long 0.1543 3.0935
pair_coeff   4 11 lj/cut/coul/long 0.0 0.0
pair_coeff   5 5 lj/cut/coul/long 0.1701 3.0700
pair_coeff   5 6 lj/cut/coul/long 0.0 0.0
pair_coeff   5 7 lj/cut/coul/long 0.1137 3.3013
pair_coeff   5 8 lj/cut/coul/long 0.1091 3.3013
pair_coeff   5 9 lj/cut/coul/long 0.0714 2.7257
pair_coeff   5 10 lj/cut/coul/long 0.1700 3.1829
pair_coeff   5 11 lj/cut/coul/long 0.0 0.0
pair_coeff   6 6 lj/cut/coul/long 0.0 0.0
pair_coeff   6 7 lj/cut/coul/long 0.0 0.0
pair_coeff   6 8 lj/cut/coul/long 0.0 0.0
pair_coeff   6 9 lj/cut/coul/long 0.0 0.0
pair_coeff   6 10 lj/cut/coul/long 0.0 0.0
pair_coeff   6 11 lj/cut/coul/long 0.0 0.0
pair_coeff   7 7 lj/cut/coul/long 0.076 3.55
pair_coeff   7 8 lj/cut/coul/long 0.0729 3.55
pair_coeff   7 9 lj/cut/coul/long 0.0477 2.9310
pair_coeff   7 10 lj/cut/coul/long 0.1137 3.4227
pair_coeff   7 11 lj/cut/coul/long 0.0 0.0
pair_coeff   8 8 lj/cut/coul/long 0.07 3.55
pair_coeff   8 9 lj/cut/coul/long 0.0458 2.9310
pair_coeff   8 10 lj/cut/coul/long 0.1091 3.4227
pair_coeff   8 11 lj/cut/coul/long 0.0 0.0
pair_coeff   9 9 lj/cut/coul/long 0.03 2.42
pair_coeff   9 10 lj/cut/coul/long 0.0714 2.8260
pair_coeff   9 11 lj/cut/coul/long 0.0 0.0
pair_coeff   10 10 lj/cut/coul/long 0.17 3.3
pair_coeff   10 11 lj/cut/coul/long 0.0 0.0
pair_coeff   11 11 lj/cut/coul/long 0.0 0.0
#neighbor    2. nsq
neigh_modify every 1 delay 0 check yes
kspace_style pppm 1.0e-4
```

```
#Fix water with shake
group water type 1 2
group go type 3 4 5 6
group an type 7 8 9 10 11
fix 1 water shake 0.0001 20 0 b 1 a 1
#Run commands
timestep 1.0

fix 2 all nve
fix 3 all langevin 300.0 300.0 1000.0 48279
fix 4 all press/berendsen x 1.0 1.0 1000.0 y 1.0 1.0 1000.0 z 1.0 1.0 1000.0 couple xy modulus 21739.13
fix 5 all momentum 1 linear 1 1 1

dump 1 all custom 5000 dump_GO_water_aniline_nve1.lammpstrj id type x y z vx vy vz
dump_modify 1 sort 1

thermo_style     multi
thermo           5000

restart 5000 restart1_GO_water_aniline_nve1 restart2_GO_water_aniline_nve1

run              20000000

write_data data_GO_water_aniline_nve1.data
write_restart restart_GO_water_aniline_nve1.restart
```

# With NaCl salt

#Atom types

**1**	**Water O**
**2**	**Water H**
**3**	**GO C**
**4**	**Epoxy O**
**5**	**Hydroxyl O**
**6**	**Hydroxyl H**
**7**	**Na**
**8**	**Cl**
**9**	**Aniline C1**
**10**	**Aniline C2**
**11**	**Aniline H1**
**12**	**Aniline N**
**13**	**Aniline H2**

```
boundary     p p p
units        real
atom_style   full

bond_style harmonic
angle_style harmonic
angle_style harmonic
dihedral_style opls
```

```
improper_style harmonic

# geometry
read_data       min.data

# potentials
pair_style hybrid lj/cut/coul/long 10.0 10.0  tersoff
pair_coeff    * * tersoff C.tersoff NULL NULL C NULL NULL NULL NULL NULL NULL NULL NULL NULL
NULL
pair_coeff   1 1 lj/cut/coul/long 0.1554 3.16557
pair_coeff   1 2 lj/cut/coul/long 0.0 0.0
pair_coeff   1 3 lj/cut/coul/long 0.0930 3.2807
pair_coeff   1 4 lj/cut/coul/long 0.1475 3.0299
pair_coeff   1 5 lj/cut/coul/long 0.1626 3.1174
pair_coeff   1 6 lj/cut/coul/long 0.0 0.0
pair_coeff   1 7 lj/cut/coul/long 0.0088 3.5894
pair_coeff   1 8 lj/cut/coul/long 0.3322 3.5673
pair_coeff   1 9 lj/cut/coul/long 0.1087 3.3523
pair_coeff   1 10 lj/cut/coul/long 0.1043 3.3523
pair_coeff   1 11 lj/cut/coul/long 0.0683 2.7678
pair_coeff   1 12 lj/cut/coul/long 0.1625 3.2321
pair_coeff   1 13 lj/cut/coul/long 0.0 0.0
pair_coeff   2 2 lj/cut/coul/long 0.0 0.0
pair_coeff   2 3 lj/cut/coul/long 0.0 0.0
pair_coeff   2 4 lj/cut/coul/long 0.0 0.0
pair_coeff   2 5 lj/cut/coul/long 0.0 0.0
pair_coeff   2 6 lj/cut/coul/long 0.0 0.0
pair_coeff   2 7 lj/cut/coul/long 0.0 0.0
pair_coeff   2 8 lj/cut/coul/long 0.0 0.0
pair_coeff   2 9 lj/cut/coul/long 0.0 0.0
pair_coeff   2 10 lj/cut/coul/long 0.0 0.0
pair_coeff   2 11 lj/cut/coul/long 0.0 0.0
pair_coeff   2 12 lj/cut/coul/long 0.0 0.0
pair_coeff   2 13 lj/cut/coul/long 0.0 0.0
pair_coeff   3 4 lj/cut/coul/long 0.0883 3.1401
pair_coeff   3 5 lj/cut/coul/long 0.0973 3.2308
pair_coeff   3 6 lj/cut/coul/long 0.0 0.0
pair_coeff   3 7 lj/cut/coul/long 0.0053 3.7199
pair_coeff   3 8 lj/cut/coul/long 0.1989 3.6970
pair_coeff   3 9 lj/cut/coul/long 0.0651 3.4742
pair_coeff   3 10 lj/cut/coul/long 0.0624 3.4742
pair_coeff   3 11 lj/cut/coul/long 0.0409 2.8684
pair_coeff   3 12 lj/cut/coul/long 0.0973 3.3496
pair_coeff   3 13 lj/cut/coul/long 0.0 0.0
pair_coeff   4 4 lj/cut/coul/long 0.1400 2.9000
pair_coeff   4 5 lj/cut/coul/long 0.1543 2.9838
pair_coeff   4 6 lj/cut/coul/long 0.0 0.0
pair_coeff   4 7 lj/cut/coul/long 0.0084 3.4355
pair_coeff   4 8 lj/cut/coul/long 0.3153 3.4144
pair_coeff   4 9 lj/cut/coul/long 0.1032 3.2086
pair_coeff   4 10 lj/cut/coul/long 0.0990 3.2086
pair_coeff   4 11 lj/cut/coul/long 0.0648 2.6492
pair_coeff   4 12 lj/cut/coul/long 0.1543 3.0935
pair_coeff   4 13 lj/cut/coul/long 0.0 0.0
pair_coeff   5 5 lj/cut/coul/long 0.1701 3.0700
pair_coeff   5 6 lj/cut/coul/long 0.0 0.0
```

```
pair_coeff   5 7 lj/cut/coul/long 0.0092 3.5348
pair_coeff   5 8 lj/cut/coul/long 0.3475 3.5130
pair_coeff   5 9 lj/cut/coul/long 0.1137 3.3013
pair_coeff   5 10 lj/cut/coul/long 0.1091 3.3013
pair_coeff   5 11 lj/cut/coul/long 0.0714 2.7257
pair_coeff   5 12 lj/cut/coul/long 0.1700 3.1829
pair_coeff   5 13 lj/cut/coul/long 0.0 0.0
pair_coeff   6 6 lj/cut/coul/long 0.0 0.0
pair_coeff   6 7 lj/cut/coul/long 0.0 0.0
pair_coeff   6 8 lj/cut/coul/long 0.0 0.0
pair_coeff   6 9 lj/cut/coul/long 0.0 0.0
pair_coeff   6 10 lj/cut/coul/long 0.0 0.0
pair_coeff   6 11 lj/cut/coul/long 0.0 0.0
pair_coeff   6 12 lj/cut/coul/long 0.0 0.0
pair_coeff   6 13 lj/cut/coul/long 0.0 0.0
pair_coeff   7 7 lj/cut/coul/long 0.0005 4.07
pair_coeff   7 8 lj/cut/coul/long 0.0188 4.0449
pair_coeff   7 9 lj/cut/coul/long 0.0062 3.8011
pair_coeff   7 10 lj/cut/coul/long 0.0059 3.8011
pair_coeff   7 11 lj/cut/coul/long 0.0039 3.1384
pair_coeff   7 12 lj/cut/coul/long 0.0092 3.6648
pair_coeff   7 13 lj/cut/coul/long 0.0 0.0
pair_coeff   8 8 lj/cut/coul/long 0.71 4.02
pair_coeff   8 9 lj/cut/coul/long 0.2323 3.7777
pair_coeff   8 10 lj/cut/coul/long 0.2229 3.7777
pair_coeff   8 11 lj/cut/coul/long 0.1459 3.1190
pair_coeff   8 12 lj/cut/coul/long 0.3474 3.6423
pair_coeff   8 13 lj/cut/coul/long 0.0 0.0
pair_coeff   9 9 lj/cut/coul/long 0.076 3.55
pair_coeff   9 10 lj/cut/coul/long 0.0729 3.55
pair_coeff   9 11 lj/cut/coul/long 0.0477 2.9310
pair_coeff   9 12 lj/cut/coul/long 0.1137 3.4227
pair_coeff   9 13 lj/cut/coul/long 0.0 0.0
pair_coeff   10 10 lj/cut/coul/long 0.07 3.55
pair_coeff   10 11 lj/cut/coul/long 0.0458 2.9310
pair_coeff   10 12 lj/cut/coul/long 0.1091 3.4227
pair_coeff   10 13 lj/cut/coul/long 0.0 0.0
pair_coeff   11 11 lj/cut/coul/long 0.03 2.42
pair_coeff   11 12 lj/cut/coul/long 0.0714 2.8260
pair_coeff   11 13 lj/cut/coul/long 0.0 0.0
pair_coeff   12 12 lj/cut/coul/long 0.17 3.3
pair_coeff   12 13 lj/cut/coul/long 0.0 0.0
pair_coeff   13 13 lj/cut/coul/long 0.0 0.0
#neighbor    2. nsq
neigh_modify every 1 delay 0 check yes
kspace_style pppm 1.0e-4

#Fix water with shake
group water type 1 2
group go type 3 4 5 6
group slt type 7 8
group an type 9 10 11 12 13
fix 1 water shake 0.0001 20 0 b 1 a 1
#Run commands
timestep 1.0
```

```
fix 2 all nve
fix 3 all langevin 300.0 300.0 1000.0 48279
fix 4 all press/berendsen x 1.0 1.0 1000.0 y 1.0 1.0 1000.0 z 1.0 1.0 1000.0 couple xy modulus 21739.13
fix 5 all momentum 1 linear 1 1 1

dump 1 all custom 5000 dump_GO_water_nacl_aniline_nve1.lammpstrj id type x y z vx vy vz
dump_modify 1 sort 1

thermo_style     multi
thermo           5000

restart 5000 restart1_GO_water_nacl_aniline_nve1 restart2_GO_water_nacl_aniline_nve1

run              20000000

write_data data_GO_water_nacl_aniline_nve1.data
write_restart restart_GO_water_nacl_aniline_nve1.restart
```

# With NaI salt

#Atom types

**1**	**Water O**
**2**	**Water H**
**3**	**GO C**
**4**	**Epoxy O**
**5**	**Hydroxyl O**
**6**	**Hydroxyl H**
**7**	**Na**
**8**	**I**
**9**	**Aniline C1**
**10**	**Aniline C2**
**11**	**Aniline H1**
**12**	**Aniline N**
**13**	**Aniline H2**

```
boundary     p p p
units        real
atom_style   full

bond_style harmonic
angle_style harmonic
angle_style harmonic
dihedral_style opls
improper_style harmonic

# geometry
read_data       min.data

# potentials
pair_style hybrid lj/cut/coul/long 10.0 10.0  tersoff
pair_coeff    * * tersoff C.tersoff NULL NULL C NULL NULL NULL NULL NULL NULL NULL NULL NULL
NULL
```

```
pair_coeff   1 1 lj/cut/coul/long 0.1554 3.16557
pair_coeff   1 2 lj/cut/coul/long 0.0 0.0
pair_coeff   1 3 lj/cut/coul/long 0.0930 3.2807
pair_coeff   1 4 lj/cut/coul/long 0.1475 3.0299
pair_coeff   1 5 lj/cut/coul/long 0.1626 3.1174
pair_coeff   1 6 lj/cut/coul/long 0.0 0.0
pair_coeff   1 7 lj/cut/coul/long 0.0088 3.5894
pair_coeff   1 8 lj/cut/coul/long 0.3322 3.9021
pair_coeff   1 9 lj/cut/coul/long 0.1087 3.3523
pair_coeff   1 10 lj/cut/coul/long 0.1043 3.3523
pair_coeff   1 11 lj/cut/coul/long 0.0683 2.7678
pair_coeff   1 12 lj/cut/coul/long 0.1625 3.2321
pair_coeff   1 13 lj/cut/coul/long 0.0 0.0
pair_coeff   2 2 lj/cut/coul/long 0.0 0.0
pair_coeff   2 3 lj/cut/coul/long 0.0 0.0
pair_coeff   2 4 lj/cut/coul/long 0.0 0.0
pair_coeff   2 5 lj/cut/coul/long 0.0 0.0
pair_coeff   2 6 lj/cut/coul/long 0.0 0.0
pair_coeff   2 7 lj/cut/coul/long 0.0 0.0
pair_coeff   2 8 lj/cut/coul/long 0.0 0.0
pair_coeff   2 9 lj/cut/coul/long 0.0 0.0
pair_coeff   2 10 lj/cut/coul/long 0.0 0.0
pair_coeff   2 11 lj/cut/coul/long 0.0 0.0
pair_coeff   2 12 lj/cut/coul/long 0.0 0.0
pair_coeff   2 13 lj/cut/coul/long 0.0 0.0
pair_coeff   3 4 lj/cut/coul/long 0.0883 3.1401
pair_coeff   3 5 lj/cut/coul/long 0.0973 3.2308
pair_coeff   3 6 lj/cut/coul/long 0.0 0.0
pair_coeff   3 7 lj/cut/coul/long 0.0053 3.7199
pair_coeff   3 8 lj/cut/coul/long 0.1989 4.0440
pair_coeff   3 9 lj/cut/coul/long 0.0651 3.4742
pair_coeff   3 10 lj/cut/coul/long 0.0624 3.4742
pair_coeff   3 11 lj/cut/coul/long 0.0409 2.8684
pair_coeff   3 12 lj/cut/coul/long 0.0973 3.3496
pair_coeff   3 13 lj/cut/coul/long 0.0 0.0
pair_coeff   4 4 lj/cut/coul/long 0.1400 2.9000
pair_coeff   4 5 lj/cut/coul/long 0.1543 2.9838
pair_coeff   4 6 lj/cut/coul/long 0.0 0.0
pair_coeff   4 7 lj/cut/coul/long 0.0084 3.4355
pair_coeff   4 8 lj/cut/coul/long 0.3153 3.7348
pair_coeff   4 9 lj/cut/coul/long 0.1032 3.2086
pair_coeff   4 10 lj/cut/coul/long 0.0990 3.2086
pair_coeff   4 11 lj/cut/coul/long 0.0648 2.6492
pair_coeff   4 12 lj/cut/coul/long 0.1543 3.0935
pair_coeff   4 13 lj/cut/coul/long 0.0 0.0
pair_coeff   5 5 lj/cut/coul/long 0.1701 3.0700
pair_coeff   5 6 lj/cut/coul/long 0.0 0.0
pair_coeff   5 7 lj/cut/coul/long 0.0092 3.5348
pair_coeff   5 8 lj/cut/coul/long 0.3475 3.8427
pair_coeff   5 9 lj/cut/coul/long 0.1137 3.3013
pair_coeff   5 10 lj/cut/coul/long 0.1091 3.3013
pair_coeff   5 11 lj/cut/coul/long 0.0714 2.7257
pair_coeff   5 12 lj/cut/coul/long 0.1700 3.1829
pair_coeff   5 13 lj/cut/coul/long 0.0 0.0
pair_coeff   6 6 lj/cut/coul/long 0.0 0.0
pair_coeff   6 7 lj/cut/coul/long 0.0 0.0
```

```
pair_coeff   6 8 lj/cut/coul/long 0.0 0.0
pair_coeff   6 9 lj/cut/coul/long 0.0 0.0
pair_coeff   6 10 lj/cut/coul/long 0.0 0.0
pair_coeff   6 11 lj/cut/coul/long 0.0 0.0
pair_coeff   6 12 lj/cut/coul/long 0.0 0.0
pair_coeff   6 13 lj/cut/coul/long 0.0 0.0
pair_coeff   7 7 lj/cut/coul/long 0.0005 4.07
pair_coeff   7 8 lj/cut/coul/long 0.0188 4.4246
pair_coeff   7 9 lj/cut/coul/long 0.0062 3.8011
pair_coeff   7 10 lj/cut/coul/long 0.0059 3.8011
pair_coeff   7 11 lj/cut/coul/long 0.0039 3.1384
pair_coeff   7 12 lj/cut/coul/long 0.0092 3.6648
pair_coeff   7 13 lj/cut/coul/long 0.0 0.0
pair_coeff   8 8 lj/cut/coul/long 0.71 4.81
pair_coeff   8 9 lj/cut/coul/long 0.2323 4.1323
pair_coeff   8 10 lj/cut/coul/long 0.2229 4.1323
pair_coeff   8 11 lj/cut/coul/long 0.1459 3.4118
pair_coeff   8 12 lj/cut/coul/long 0.3474 3.9841
pair_coeff   8 13 lj/cut/coul/long 0.0 0.0
pair_coeff   9 9 lj/cut/coul/long 0.076 3.55
pair_coeff   9 10 lj/cut/coul/long 0.0729 3.55
pair_coeff   9 11 lj/cut/coul/long 0.0477 2.9310
pair_coeff   9 12 lj/cut/coul/long 0.1137 3.4227
pair_coeff   9 13 lj/cut/coul/long 0.0 0.0
pair_coeff   10 10 lj/cut/coul/long 0.07 3.55
pair_coeff   10 11 lj/cut/coul/long 0.0458 2.9310
pair_coeff   10 12 lj/cut/coul/long 0.1091 3.4227
pair_coeff   10 13 lj/cut/coul/long 0.0 0.0
pair_coeff   11 11 lj/cut/coul/long 0.03 2.42
pair_coeff   11 12 lj/cut/coul/long 0.0714 2.8260
pair_coeff   11 13 lj/cut/coul/long 0.0 0.0
pair_coeff   12 12 lj/cut/coul/long 0.17 3.3
pair_coeff   12 13 lj/cut/coul/long 0.0 0.0
pair_coeff   13 13 lj/cut/coul/long 0.0 0.0
#neighbor    2. nsq
neigh_modify every 1 delay 0 check yes
kspace_style pppm 1.0e-4

#Fix water with shake
group water type 1 2
group go type 3 4 5 6
group slt type 7 8
group an type 9 10 11 12 13
fix 1 water shake 0.0001 20 0 b 1 a 1
#Run commands
timestep 1.0

fix 2 all nve
fix 3 all langevin 300.0 300.0 1000.0 48279
fix 4 all press/berendsen x 1.0 1.0 1000.0 y 1.0 1.0 1000.0 z 1.0 1.0 1000.0 couple xy modulus 21739.13
fix 5 all momentum 1 linear 1 1 1

dump 1 all custom 5000 dump_GO_water_naI_aniline_nve1.lammpstrj id type x y z vx vy vz
dump_modify 1 sort 1

thermo_style     multi
```

thermo 5000

restart 5000 restart1_GO_water_naI_aniline_nve1 restart2_GO_water_naI_aniline_nve1

run 20000000

write_data data_GO_water_naI_aniline_nve1.data
write_restart restart_GO_water_naI_aniline_nve1.restart

``````````````````````````````````````````````````````````````````````````````````````````````````````````

**Tersoff File Parameters**

# Use SiC.tersoff vallues and converted them from metal units to real units by Visal
# values are from Phys Rev B, 39, 5566-5568 (1989)
# and errata (PRB 41, 3248)
# Tersoff parameters for various elements and mixtures
# multiple entries can be added to this file, LAMMPS reads the ones it needs
# these entries are in LAMMPS "metal" units:
# A,B = Kcal/mol; lambda1,lambda2,lambda3 = 1/Angstroms; R,D = Angstroms
# other quantities are unitless

# format of a single entry (one or more lines):
# element 1, element 2, element 3, m, gamma, lambda3, c, d, costheta0, n, beta, lambda2, B, R, D, lambda1, A

C C C 3.0 1.0 0.0 38049 4.3484 -.57058 .72751 0.00000015724 2.2119 7994.02 1.95 0.15 3.4879 32132.89

Drude Oscillator Model

units real
boundary p p p

atom_style full
bond_style harmonic
angle_style harmonic
dihedral_style opls
improper_style harmonic
special_bonds lj/coul 0.0 0.0 1.0

pair_style hybrid lj/cut/coul/long 10.0 10.0 lj/cut/thole/long 0.50 10.0 10.0
kspace_style pppm 1.0e-4

read_data data_GO4_water_p_final.data extra/special/per/atom 9

fix DRUDE all drude C N N D C C C C C N D D D D D

pair_coeff 1 1 lj/cut/coul/long 0.21093900501728058 3.1839499473571777
pair_coeff 1 2 lj/cut/coul/long 0.0000000000000000 0.0000000000000000
pair_coeff 1 3 lj/cut/coul/long 0.0000000000000000 0.0000000000000000
pair_coeff 1 4 lj/cut/coul/long 0.0000000000000000 0.0000000000000000
``````````````````````````````````````````````````````````````````````````````````````````````````````````

pair_coeff	1	5	lj/cut/coul/long	0.11799141602550099	3.3382367824571886
pair_coeff	1	6	lj/cut/coul/long	0.17184720197940007	3.0386600913858306
pair_coeff	1	7	lj/cut/coul/long	0.12661502227548910	3.3619967521244600
pair_coeff	1	8	lj/cut/coul/long	0.12151432184756808	3.3619967521244600
pair_coeff	1	9	lj/cut/coul/long	0.18936639414142636	3.1264558410179002
pair_coeff	1	10	lj/cut/coul/long	7.9548469622919310E-002	2.7758168375309769
pair_coeff	1	11	lj/cut/coul/long	0.0000000000000000	0.0000000000000000
pair_coeff	1	12	lj/cut/coul/long	0.0000000000000000	0.0000000000000000
pair_coeff	1	13	lj/cut/coul/long	0.0000000000000000	0.0000000000000000
pair_coeff	1	14	lj/cut/coul/long	0.0000000000000000	0.0000000000000000
pair_coeff	1	15	lj/cut/coul/long	0.0000000000000000	0.0000000000000000
pair_coeff	2	2	lj/cut/coul/long	0.0000000000000000	0.0000000000000000
pair_coeff	2	3	lj/cut/coul/long	0.0000000000000000	0.0000000000000000
pair_coeff	2	4	lj/cut/coul/long	0.0000000000000000	0.0000000000000000
pair_coeff	2	5	lj/cut/coul/long	0.0000000000000000	0.0000000000000000
pair_coeff	2	6	lj/cut/coul/long	0.0000000000000000	0.0000000000000000
pair_coeff	2	7	lj/cut/coul/long	0.0000000000000000	0.0000000000000000
pair_coeff	2	8	lj/cut/coul/long	0.0000000000000000	0.0000000000000000
pair_coeff	2	9	lj/cut/coul/long	0.0000000000000000	0.0000000000000000
pair_coeff	2	10	lj/cut/coul/long	0.0000000000000000	0.0000000000000000
pair_coeff	2	11	lj/cut/coul/long	0.0000000000000000	0.0000000000000000
pair_coeff	2	12	lj/cut/coul/long	0.0000000000000000	0.0000000000000000
pair_coeff	2	13	lj/cut/coul/long	0.0000000000000000	0.0000000000000000
pair_coeff	2	14	lj/cut/coul/long	0.0000000000000000	0.0000000000000000
pair_coeff	2	15	lj/cut/coul/long	0.0000000000000000	0.0000000000000000
pair_coeff	3	3	lj/cut/coul/long	0.0000000000000000	0.0000000000000000
pair_coeff	3	4	lj/cut/coul/long	0.0000000000000000	0.0000000000000000
pair_coeff	3	5	lj/cut/coul/long	0.0000000000000000	0.0000000000000000
pair_coeff	3	6	lj/cut/coul/long	0.0000000000000000	0.0000000000000000
pair_coeff	3	7	lj/cut/coul/long	0.0000000000000000	0.0000000000000000
pair_coeff	3	8	lj/cut/coul/long	0.0000000000000000	0.0000000000000000
pair_coeff	3	9	lj/cut/coul/long	0.0000000000000000	0.0000000000000000
pair_coeff	3	10	lj/cut/coul/long	0.0000000000000000	0.0000000000000000
pair_coeff	3	11	lj/cut/coul/long	0.0000000000000000	0.0000000000000000
pair_coeff	3	12	lj/cut/coul/long	0.0000000000000000	0.0000000000000000
pair_coeff	3	13	lj/cut/coul/long	0.0000000000000000	0.0000000000000000
pair_coeff	3	14	lj/cut/coul/long	0.0000000000000000	0.0000000000000000
pair_coeff	3	15	lj/cut/coul/long	0.0000000000000000	0.0000000000000000
pair_coeff	4	4	lj/cut/coul/long	0.0000000000000000	0.0000000000000000
pair_coeff	4	5	lj/cut/coul/long	0.0000000000000000	0.0000000000000000
pair_coeff	4	6	lj/cut/coul/long	0.0000000000000000	0.0000000000000000
pair_coeff	4	7	lj/cut/coul/long	0.0000000000000000	0.0000000000000000
pair_coeff	4	8	lj/cut/coul/long	0.0000000000000000	0.0000000000000000
pair_coeff	4	9	lj/cut/coul/long	0.0000000000000000	0.0000000000000000
pair_coeff	4	10	lj/cut/coul/long	0.0000000000000000	0.0000000000000000
pair_coeff	4	11	lj/cut/coul/long	0.0000000000000000	0.0000000000000000
pair_coeff	4	12	lj/cut/coul/long	0.0000000000000000	0.0000000000000000
pair_coeff	4	13	lj/cut/coul/long	0.0000000000000000	0.0000000000000000
pair_coeff	4	14	lj/cut/coul/long	0.0000000000000000	0.0000000000000000
pair_coeff	4	15	lj/cut/coul/long	0.0000000000000000	0.0000000000000000

pair_coeff 5 5 lj/cut/thole/long 6.5999999642372131E-002 3.5000000000000000
1.5620000362396240
pair_coeff 5 6 lj/cut/thole/long 9.6124918669776585E-002 3.1859065167995766
0.98250495009546313
pair_coeff 5 7 lj/cut/thole/long 7.0823723507946926E-002 3.5249113227295514
1.5882789658694678
pair_coeff 5 8 lj/cut/thole/long 6.7970581832404398E-002 3.5249113227295514
1.3051885921098683
pair_coeff 5 9 lj/cut/thole/long 0.10592450168502308 3.2779566449771407
0.99089516340817951
pair_coeff 5 10 lj/cut/coul/long 4.4496448500365667E-002 2.9103264880471413
pair_coeff 5 11 lj/cut/thole/long 0.0000000000000000 0.0000000000000000
1.5620000362396240
pair_coeff 5 12 lj/cut/thole/long 0.0000000000000000 0.0000000000000000
0.98250495009546313
pair_coeff 5 13 lj/cut/thole/long 0.0000000000000000 0.0000000000000000
1.5882789658694678
pair_coeff 5 14 lj/cut/thole/long 0.0000000000000000 0.0000000000000000
1.3051885921098683
pair_coeff 5 15 lj/cut/thole/long 0.0000000000000000 0.0000000000000000
0.99089516340817951
pair_coeff 6 6 lj/cut/thole/long 0.14000000059604645 2.9000000953674316
0.61799997091293335
pair_coeff 6 7 lj/cut/thole/long 0.10315037422699380 3.2085822726356263
0.99903451337683746
pair_coeff 6 8 lj/cut/thole/long 9.8994949787585135E-002 3.2085822726356263
0.82096941280694691
pair_coeff 6 9 lj/cut/thole/long 0.15427248734517574 2.9837895534340424
0.62327745229631792
pair_coeff 6 10 lj/cut/coul/long 6.4806326005577286E-002 2.6491508926525182
pair_coeff 6 11 lj/cut/thole/long 0.0000000000000000 0.0000000000000000
0.98250495009546313
pair_coeff 6 12 lj/cut/thole/long 0.0000000000000000 0.0000000000000000
0.61799997091293335
pair_coeff 6 13 lj/cut/thole/long 0.0000000000000000 0.0000000000000000
0.99903451337683746
pair_coeff 6 14 lj/cut/thole/long 0.0000000000000000 0.0000000000000000
0.82096941280694691
pair_coeff 6 15 lj/cut/thole/long 0.0000000000000000 0.0000000000000000
0.62327745229631792
pair_coeff 7 7 lj/cut/thole/long 7.5999997556209564E-002 3.5499999523162842
1.6150000095367432
pair_coeff 7 8 lj/cut/thole/long 7.2938329097837401E-002 3.5499999523162842
1.3271469521418575
pair_coeff 7 9 lj/cut/thole/long 0.11366617667738373 3.3012875695132844
1.0075658827843736
pair_coeff 7 10 lj/cut/coul/long 4.7748548223510746E-002 2.9310407973020283
pair_coeff 7 11 lj/cut/thole/long 0.0000000000000000 0.0000000000000000
1.5882789658694678
pair_coeff 7 12 lj/cut/thole/long 0.0000000000000000 0.0000000000000000
0.99903451337683746

```
pair_coeff 7 13 lj/cut/thole/long 0.0000000000000000 0.0000000000000000
1.6150000095367432
pair_coeff 7 14 lj/cut/thole/long 0.0000000000000000 0.0000000000000000
1.3271469521418575
pair_coeff 7 15 lj/cut/thole/long 0.0000000000000000 0.0000000000000000
1.0075658827843736
pair_coeff 8 8 lj/cut/thole/long 7.0000000298023224E-002 3.5499999523162842
1.0906000137329102
pair_coeff 8 9 lj/cut/thole/long 0.10908712195228959 3.3012875695132844
0.82798017493694587
pair_coeff 8 10 lj/cut/coul/long 4.5824992582329797E-002 2.9310407973020283
pair_coeff 8 11 lj/cut/thole/long 0.0000000000000000 0.0000000000000000
1.3051885921098683
pair_coeff 8 12 lj/cut/thole/long 0.0000000000000000 0.0000000000000000
0.82096941280694691
pair_coeff 8 13 lj/cut/thole/long 0.0000000000000000 0.0000000000000000
1.3271469521418575
pair_coeff 8 14 lj/cut/thole/long 0.0000000000000000 0.0000000000000000
1.0906000137329102
pair_coeff 8 15 lj/cut/thole/long 0.0000000000000000 0.0000000000000000
0.82798017493694587
pair_coeff 9 9 lj/cut/thole/long 0.17000000178813934 3.0699999332427979
0.62860000133514404
pair_coeff 9 10 lj/cut/coul/long 7.1413093328694527E-002 2.7256925858706036
pair_coeff 9 11 lj/cut/thole/long 0.0000000000000000 0.0000000000000000
0.99089516340817951
pair_coeff 9 12 lj/cut/thole/long 0.0000000000000000 0.0000000000000000
0.62327745229631792
pair_coeff 9 13 lj/cut/thole/long 0.0000000000000000 0.0000000000000000
1.0075658827843736
pair_coeff 9 14 lj/cut/thole/long 0.0000000000000000 0.0000000000000000
0.82798017493694587
pair_coeff 9 15 lj/cut/thole/long 0.0000000000000000 0.0000000000000000
0.62860000133514404
pair_coeff 10 10 lj/cut/coul/long 2.9998999089002609E-002 2.4200000762939453
pair_coeff 10 11 lj/cut/coul/long 0.0000000000000000 0.0000000000000000
pair_coeff 10 12 lj/cut/coul/long 0.0000000000000000 0.0000000000000000
pair_coeff 10 13 lj/cut/coul/long 0.0000000000000000 0.0000000000000000
pair_coeff 10 14 lj/cut/coul/long 0.0000000000000000 0.0000000000000000
pair_coeff 10 15 lj/cut/coul/long 0.0000000000000000 0.0000000000000000
pair_coeff 11 11 lj/cut/thole/long 0.0000000000000000 0.0000000000000000
1.5620000362396240
pair_coeff 11 12 lj/cut/thole/long 0.0000000000000000 0.0000000000000000
0.98250495009546313
pair_coeff 11 13 lj/cut/thole/long 0.0000000000000000 0.0000000000000000
1.5882789658694678
pair_coeff 11 14 lj/cut/thole/long 0.0000000000000000 0.0000000000000000
1.3051885921098683
pair_coeff 11 15 lj/cut/thole/long 0.0000000000000000 0.0000000000000000
0.99089516340817951
```

```
 pair_coeff         12         12 lj/cut/thole/long    0.0000000000000000        0.0000000000000000
0.61799997091293335
 pair_coeff         12         13 lj/cut/thole/long    0.0000000000000000        0.0000000000000000
0.99903451337683746
 pair_coeff         12         14 lj/cut/thole/long    0.0000000000000000        0.0000000000000000
0.82096941280694691
 pair_coeff         12         15 lj/cut/thole/long    0.0000000000000000        0.0000000000000000
0.62327745229631792
 pair_coeff         13         13 lj/cut/thole/long    0.0000000000000000        0.0000000000000000
1.6150000095367432
 pair_coeff         13         14 lj/cut/thole/long    0.0000000000000000        0.0000000000000000
1.3271469521418575
 pair_coeff         13         15 lj/cut/thole/long    0.0000000000000000        0.0000000000000000
1.0075658827843736
 pair_coeff         14         14 lj/cut/thole/long    0.0000000000000000        0.0000000000000000
1.0906000137329102
 pair_coeff         14         15 lj/cut/thole/long    0.0000000000000000        0.0000000000000000
0.82798017493694587
 pair_coeff         15         15 lj/cut/thole/long    0.0000000000000000        0.0000000000000000
0.62860000133514404

group graphene type 5 6 7 8 9 10 11 12 13 14 15
group core_graphene type 5 6 7 8 9
group drude_graphene type 11 12 13 14 15

group water type 1 2 3 4

group water_atoms type 1 2 3
group water_drude type 4

# delete bonds in water molecules
delete_bonds water_atoms multi

reset_timestep 0

variable TK equal 300.0
variable TDRUDE equal 1.0
variable PBAR equal 1.0

neighbor 2.0 bin
neigh_modify one 10000

# Set the charges now for the interacting drude particles
variable lambda equal 1.0
variable q1 atom 2.3282*v_lambda
variable q2 atom -2.3282*v_lambda

timestep 0.05

velocity water_atoms create ${TK} 12345
run 0
```

```
velocity water_atoms scale ${TK}

velocity water_drude create ${TDRUDE} 12356

velocity drude_graphene create ${TDRUDE} 12396
velocity core_graphene create ${TK} 12857

comm_modify vel yes

# Thermostats for graphene
fix nvt_start graphene drude/transform/direct
fix nvt_graphene core_graphene nvt temp ${TK} ${TK} 100.0
fix nvt_drude_graphene drude_graphene nvt temp ${TDRUDE} ${TDRUDE} 5.0
fix nvt_end graphene drude/transform/inverse

fix momentum_graphene graphene momentum 100 linear 1 1 1

# Thermostats for water
fix DTDIR water drude/transform/direct
fix RIGID water_atoms rigid/nvt/small molecule temp ${TK} ${TK} 100.0
fix NVT water_drude nvt temp ${TDRUDE} ${TDRUDE} 5.0
fix DTINV water drude/transform/inverse

fix momentum_water water momentum 100 linear 1 1 1

#Equilibrate the system for 500 ps of simulation time
run 1000

timestep 0.5

compute tgraphene graphene temp/drude
compute twater water temp/drude
compute watertemp water_atoms temp/com

dump 1 all custom 100 dump_graphene-p_nvt2.lammpstrj id type element x y z
dump_modify 1 sort 1

thermo_style custom step etotal ke pe ebond eangle evdwl ecoul elong press vol temp c_twater[1]
c_twater[2] c_tgraphene[1] c_tgraphene[2] c_watertemp
thermo 1000

restart 2000 restart1_graphene-p_pol_nvt2 restart2_graphene-p_pol_nvt2

run 5000000

write_data data_graphene-p_pol_nvt2.data
write_restart restart_graphene-p_pol_nvt2.restart

Masses

  1   15.599  # Ow DC
```

```
  2    1.008  # Hw
  3    1.e-16 # M
  4    0.400  # Ow DP
  5   11.611  # SP3COe DC
  6   15.599  # Oe DC
  7   11.611  # Sp2C DC
  8   11.611  # Sp3COh DC
  9   15.599  # Oh DC
 10    1.008  # Ho
 11    0.400  # SP3COe DP
 12    0.400  # Oe DP
 13    0.400  # Sp2C DP
 14    0.400  # Sp3COh DP
 15    0.400  # Oh DP

Bond Coeffs

  1   517.630258     0.957200  # Ow-Hw
  2   500.000000     0.240340  # Ow-M
  3   500.000000     0.000000  # Ow DC-DP
  4   553.0 0.945  #63
  5   317.0 1.510  #127
  6   320.0 1.410  #119
  7   268.0 1.529  #113
  8   549.0 1.340  #261
  9   450.0 1.364  #71
 10   469.0 1.400  #272
 11   427.0 1.433  #262
 12   317.0 1.510  #126
 13   500.000000     0.000000  # SP3COe DC-DP
 14   500.000000     0.000000  # Oe DC-DP
 15   500.000000     0.000000  # Sp2C DC-DP
 16   500.000000     0.000000  # Sp3COh DC-DP
 17   500.000000     0.000000  # Oh DC-DP
```

#Atom types

**1**	**Water O drude core**
**2**	**Water H**
**3**	**Polarizable water massless site**
**4**	**Water O drude particle**
**5**	**SP3 C (connected to epoxy) drude core**
**6**	**Epoxy O drude core**
**7**	**SP2 C drude core**
**8**	**SP3 C (connected to hydroxy) drude core**
**9**	Hydroxy O drude core
**10**	**Hydroxy H**
**11**	**SP3 C (connected to epoxy) drude particle**
**12**	**Epoxy O drude particle**
**13**	**SP2 C drude particle**

14	Sp3 C (connected to hydroxy) drude particle
15	Hydroxyl O drude particle

References

1. David, R.; Tuladhar, A.; Zhang, L.; Arges, C.; Kumar, R., Effect of Oxidation Level on the Interfacial Water at the Graphene Oxide–Water Interface: From Spectroscopic Signatures to Hydrogen-Bonding Environment. *J. Phys. Chem. B* **2020,** *124* (37), 8167-8178.
2. Jorgensen, W. L.; Maxwell, D. S.; Tirado-Rives, J., Development and Testing of the OPLS All-Atom Force Field on Conformational Energetics and Properties of Organic Liquids. *J. Amer. Chem Soc.* **1996,** *118* (45), 11225-11236.
3. Berendsen, H. J. C.; Grigera, J. R.; Straatsma, T. P., The missing term in effective pair potentials. *J. Phys. Chem.* **1987,** *91* (24), 6269-6271.
4. Lamoureux, G.; Harder, E.; Vorobyov, I. V.; Roux, B.; MacKerell, A. D., A polarizable model of water for molecular dynamics simulations of biomolecules. *Chem. Phys. Letts.* **2006,** *418* (1), 245-249.
5. Lamoureux, G.; Roux, B. t., Modeling induced polarization with classical Drude oscillators: Theory and molecular dynamics simulation algorithm. *J. Chem. Phys.* **2003,** *119* (6), 3025-3039.
6. Tainter, C. J.; Shi, L.; Skinner, J. L., Reparametrized E3B (Explicit Three-Body) Water Model Using the TIP4P/2005 Model as a Reference. *J. Chem. Theor. Comput.* **2015,** *11* (5), 2268-2277.
7. Tersoff, J., Empirical interatomic potential for silicon with improved elastic properties. *Phys. Rev. B* **1988,** *38* (14), 9902-9905.
8. Tersoff, J., Modeling solid-state chemistry: Interatomic potentials for multicomponent systems. *Phys. Rev.B* **1989,** *39* (8), 5566.
9. Willard, A. P.; Chandler, D., Instantaneous Liquid Interfaces. *J. Phys. Chem. B* **2010,** *114* (5), 1954-1958.
10. Pezzotti, S.; Galimberti, D. R.; Gaigeot, M.-P., 2D H-Bond Network as the Topmost Skin to the Air–Water Interface. *J. Phys. Chem. Letts.* **2017,** *8* (13), 3133-3141.
11. Pezzotti, S.; Galimberti, D. R.; Shen, Y. R.; Gaigeot, M.-P., Structural definition of the BIL and DL: a new universal methodology to rationalize non-linear χ(2)(ω) SFG signals at charged interfaces, including χ(3)(ω) contributions. *Phys. Chem. Chem. Phys.* **2018,** *20* (7), 5190-5199.
